# Exploratory Single-Nucleus Transcriptomic and Chromatin Accessibility Profiling Indicates Microglial Remodeling in Tuberous Sclerosis Complex-Associated Epilepsy

**DOI:** 10.3390/biology15211976

**Published:** 2026-11-02

**Authors:** Jiaqi Zhang, Yuanyuan Dang, Lin Wan, Qiyan Liu, Xinting Liu, Linyan Hu, Jing Wang, Xiuyu Shi, Hulin Zhao, Guang Yang

**Affiliations:** 1 Senior Department of Pediatrics, Chinese PLA General Hospital, Beijing 100700, China; 15620949010@163.com (J.Z.);; 2 Department of Pediatrics, The First Medical Center, Chinese PLA General Hospital, Beijing 100853, China; 3 Medical School of Chinese People’s Liberation Army, Beijing 100853, China; 4 Senior Department of Neurosurgery, The First Medical Center of Chinese PLA General Hospital, Beijing 100853, China; dyy419@163.com; 5 Guizhou University of Traditional Chinese Medicine, Guiyang 550025, China

**Keywords:** tuberous sclerosis complex, epilepsy, single-nucleus RNA sequencing, single-nucleus ATAC sequencing, microglia, lipid transport, lysosome

## Abstract

Background: Tuberous sclerosis complex (TSC) frequently causes epilepsy, but cell-type-specific molecular alterations in human lesions remain incompletely understood. Methods: We performed single-nucleus RNA sequencing of cortical tissue from three TSC and three surgical control donors, with single-nucleus chromatin accessibility profiling in two donors per group from the same cohort. Donor-level pseudobulk differential analyses were combined with functional enrichment, inferred cell communication, and motif analyses. Results: Among the six cell types displayed in the main comparison, microglia had the most differentially expressed genes (872; 282 upregulated and 590 downregulated). Downregulated genes were enriched for lysosomal and lipid-storage processes. Inferred APP-CD74 input to microglia was higher for most source cell types, whereas APP-SORL1 changes varied by source. Chromatin analysis identified 9283 differentially accessible regions. Predicted regions linked to ABCA1, ATP6V0A1, and GPNMB showed decreased accessibility, concordant with lower RNA expression. Donor-averaged TFEB- and TFE3-associated chromatin variation across regions (chromVAR) scores were lower in TSC. Conclusions: These exploratory findings suggest microglial remodeling involving lysosomal and lipid-storage processes. It may provide a molecular basis for further investigation of microglia in epileptogenesis.

## Simple Summary

Tuberous sclerosis complex (TSC) frequently causes epilepsy, but the cellular changes associated with its brain lesions remain incompletely understood. We profiled gene expression in brain-tissue nuclei from six surgical donors and chromatin accessibility in four donors from the same cohort. After excluding RNA nuclei with supported or uncertain border-associated macrophage-like features, microglia showed differences involving lipid transport and intracellular material processing. Predicted cell communication and chromatin analyses provided complementary observations for selected genes and regulatory motifs. These findings nominate candidates for further investigation and suggest possible alterations in lipid clearance or lysosomal function, although these functions were not directly measured. Additional donors and functional experiments are needed.

## 1. Introduction

Tuberous sclerosis complex (TSC) is an autosomal dominant multisystem disorder caused by pathogenic variants in TSC1 or TSC2, which encode hamartin and tuberin, respectively. The TSC1-TSC2 complex negatively regulates mechanistic target of rapamycin complex 1 (mTORC1), linking these proteins to cell growth and metabolism [1,2]. Epilepsy is a major manifestation of TSC, and drug resistance imposes a substantial clinical burden [3,4,5]. Adjunctive everolimus reduces seizure frequency in some patients with treatment-resistant TSC-associated seizures [6]. Nevertheless, incomplete treatment responses motivate further investigation of the cellular alterations associated with epileptogenic lesions.

Cortical tubers are characteristic TSC-associated malformations with disorganized cortical architecture, dysmorphic neurons, giant cells, and glial abnormalities [7]. Their cellular heterogeneity complicates the interpretation of bulk molecular profiles, which average signals across distinct populations. Cell-type-resolved analyses can help identify the populations and molecular processes associated with lesion pathology, although cross-sectional profiling alone cannot determine which changes initiate seizures.

Glial dysfunction is a plausible contributor to TSC-associated epilepsy. Astrocyte-targeted Tsc1 deletion produces seizures in mice [8], and studies in related models implicate reduced glutamate transport and inward-rectifier potassium currents [9,10]. Human TSC-associated lesions also show microglial activation and inflammatory signaling [11], while cortical tubers exhibit abnormalities of oligodendroglia and myelin [12]. These observations motivate investigation of glial states in human lesions, without assuming that mechanisms established in experimental models apply uniformly across glial populations.

One candidate theme is altered lipid handling and endosomal-lysosomal processing. mTOR signaling connects nutrient availability with anabolic and catabolic processes [2], and lipid-droplet-accumulating microglia have been associated with dysfunction in the aging brain [13]. Whether related transcriptional features occur in TSC lesions, and whether they coincide with changes in chromatin accessibility, remains an open question. Single-nucleus chromatin profiling provides a complementary approach to examine cell-type-associated regulatory features in human brain tissue [14].

Here, we analyzed single-nucleus RNA sequencing (snRNA-seq) data from three TSC and three control donors and single-nucleus assay for transposase-accessible chromatin using sequencing (snATAC-seq) data from two donors per group drawn from the same cohort. Differential expression and accessibility were tested using donor-level pseudobulk profiles. Cell-type proportions, pooled ligand-receptor networks, chromatin variation across regions (chromVAR) scores, and footprints were interpreted descriptively. We combined cell communication inference [15], genomic region enrichment [16], and motif-associated accessibility analysis [17] to investigate microglial molecular changes and nominate candidates for subsequent validation.

## 2. Materials and Methods

### 2.1. Participants and Tissue Collection

Three children with TSC-associated epilepsy and three pediatric surgical controls were included (clinical details in Table S1). All participants were minors, and written informed consent was obtained from their legal guardians. The study was conducted in accordance with the Declaration of Helsinki and approved by the Ethics Committee of Chinese PLA General Hospital (approval no. S2025-280-02). Resected cortical tissue was washed in calcium- and magnesium-free phosphate-buffered saline (Thermo Fisher Scientific, Waltham, MA, USA) and placed in tissue preservation solution (Thermo Fisher Scientific, Waltham, MA, USA) before processing by Shanghai Applied Protein Technology Co., Ltd. (Shanghai, China).

### 2.2. Single-Nucleus RNA Sequencing

Single-nucleus RNA-seq libraries were prepared from nuclei isolated from cryopreserved tissue using the Chromium Single Cell 3′ v3 workflow on the Chromium X platform (10× Genomics) and sequenced on an Illumina NovaSeq 6000 platform by Shanghai Applied Protein Technology Co., Ltd. Six independent donors were processed as separate libraries. The analysis retained neuronal, glial, and vascular populations.

### 2.3. Nuclei Capture and cDNA Synthesis

Nuclear suspensions were assessed and counted before loading onto the Chromium X instrument (10× Genomics, Pleasanton, CA, USA), where individual nuclei were partitioned into gel bead-in-emulsions (GEMs). Barcoded oligo(dT) primers captured polyadenylated RNA during reverse transcription, incorporating a 16-bp nucleus barcode and a 12-bp unique molecular identifier (UMI). GEM reverse transcription was performed at 53 °C for 45 min, followed by enzyme inactivation at 85 °C for 5 min.

Following emulsion breakage, first-strand cDNA was recovered and purified using magnetic beads. Barcoded cDNA was amplified for 13 cycles using a Bio-Rad C1000 Touch thermal cycler (Bio-Rad Laboratories, Hercules, CA, USA) and assessed for quality before library construction.

### 2.4. Single-Nucleus RNA-Seq Library Preparation

Amplified cDNA underwent fragmentation, end repair, A-tailing, adapter ligation, and size selection. Sample-index polymerase chain reaction (PCR) produced gene-expression libraries, which were quality-checked and quantified using Qubit (Invitrogen, Thermo Fisher Scientific, Waltham, MA, USA) before sequencing. Libraries were sequenced on an Illumina NovaSeq 6000 instrument (Illumina, San Diego, CA, USA) in paired-end 150-bp mode (PE150). Read 1 encoded the 16-bp barcode and 12-bp UMI, whereas Read 2 contained the transcript sequence used for gene-level quantification. Sequencing depth exceeded 20,000 mean reads per nucleus in each library, as reported by the Cell Ranger “Mean Reads per Cell” metric.

### 2.5. Raw Data Processing, Quality Control, and Clustering

Cell Ranger-generated gene-barcode matrices were analyzed using Seurat (v5.5.1) [18]. Genes detected in fewer than three nuclei within a library were excluded during object construction. Nuclei were retained if they had more than 450 and fewer than 5500 detected genes, fewer than 20,000 UMIs, and less than 5% mitochondrial counts. Doublets were assessed separately within each library using DoubletFinder and Scrublet; nuclei classified as doublets by either method were subsequently excluded. The selected pooled RNA workflow used log normalization (LogNormalize; scale.factor = 10,000), variable-feature selection (vst; 2000 features), scaling without covariate regression, and principal component analysis (PCA; 50 components), without Harmony or LIGER integration. A nearest-neighbor graph was constructed from PCs 1–30, followed by Louvain clustering at resolution 1 and uniform manifold approximation and projection (UMAP) visualization (seed 20260929). Cell-type labels were assigned using canonical markers; unresolved nuclei were labeled Unknown. Normalization, variable-feature selection, PCA, neighbor construction, clustering, and UMAP were repeated after doublet removal on 50,148 singlets. Microglial identity was then reassessed using joint resident-microglial and macrophage-associated marker evidence. Nineteen nuclei were classified as supported border-associated macrophage-like (BAM-like) candidates and 90 as uncertain BAM-like candidates; these are operational transcriptomic classifications, not lineage-validated identities. The strict primary RNA analysis excluded all 109 candidates, retaining 6520 microglial nuclei and 50,039 nuclei in the displayed atlas. Marker-expression evidence is provided in Supplementary Figure S7; donor quality Control (QC) and exclusion counts are provided in Supplementary Figure S2 and Table S2.

For annotation support, each final population was compared with the remaining nuclei using a pooled Wilcoxon test (Seurat FindMarkers; only.pos = TRUE, min.pct = 0.1, logfc.threshold = 0.25). Positive markers with Seurat Bonferroni-adjusted *p* < 0.05 were ordered by decreasing avg_log2FC, then adjusted *p* value and gene name. The top five per population were displayed; duplicate genes were shown once. This analysis was not used to infer disease-associated changes. The microglial expression heatmap displays log2 (DESeq2 size-factor-normalized pseudobulk counts + 1), standardized within each gene across six donors.

### 2.6. Differential Gene Expression Analysis and Functional Enrichment Analysis

Raw RNA UMI counts were summed within each independent donor and annotated population to construct pseudobulk profiles (three TSC and three Control donors), avoiding nucleus-level pseudoreplication [19]. Differential expression was tested using DESeq2 (v1.46.0) with design ~group, local dispersion fitting (fitType = “local”), and the negative binomial Wald test [20]. Automatic outlier replacement was disabled (minReplicatesForReplace = Inf), and results were extracted with independentFiltering = FALSE. Valid gene-level *p* values were adjusted by the Benjamini-Hochberg (BH) method within each tested population. Differentially expressed genes (DEGs) required Benjamini–Hochberg false discovery rate (BH-FDR) < 0.05 and an absolute DESeq2 model log2 fold change of at least 0.25; both significance and effect-size filtering therefore came from the same donor-level model. Positive coefficients denote higher expression in TSC. The strict microglial analysis used 6520 nuclei and the fixed 27,230-gene test universe used for population comparisons. Each donor was omitted in turn, retaining the same test universe and thresholds, to assess sensitivity rather than independent replication. All annotated populations were assessed for donor coverage; populations with at least 10 nuclei in every donor were eligible for the complete 3-versus-3 comparison. The microglial results and the cell-type coverage assessment are provided in Supplementary Table S3. Complete over-representation analysis (ORA) results are provided in Supplementary Table S4; donor-omission results are shown in Supplementary Figures S3 and S4 and Table S5. State coverage and state-restricted sensitivity results are provided in Supplementary Figure S5 and Table S6. Preranked gene set enrichment analysis (GSEA) was additionally performed using fgsea (v1.32.4) on all genes with a finite DESeq2 Wald statistic, without a DEG significance threshold. Gene symbols were mapped to Entrez identifiers using org.Hs.eg.db (v3.20.0); one-to-many symbol mappings were excluded, and duplicate Entrez identifiers were represented by the gene with the highest baseMean. Gene Ontology (GO) Biological Process annotations (GO.db v3.20.0) and the archived human Kyoto Encyclopedia of Genes and Genomes (KEGG) gene sets were tested separately. Gene sets with 15–500 ranked genes were analyzed using fgseaMultilevel with scoreType = “std”, gseaParam = 1, eps = 0, nPermSimple = 10,000, and random seed 20261022. BH correction was applied across all tested terms within each database. Negative normalized enrichment scores (NES) indicate enrichment toward negative TSC-versus-Control Wald statistics. Selected plots and complete results are provided in Supplementary Figure S6 and Table S7. Upregulated and downregulated microglial DEGs were analyzed separately by ORA using clusterProfiler (v4.14.0) [21]. Gene symbols were mapped to Entrez Gene identifiers using org.Hs.eg.db (v3.20.0); unmapped or ambiguously mapped symbols were excluded, and Entrez identifiers were deduplicated. The background comprised genes tested in the same microglial differential analysis with valid *p* values and unambiguous mappings, further restricted to genes annotated in each database. GO Biological Process analysis used enrichGO with GO.db (v3.20.0). KEGG analysis used enricher with human pathway-gene mappings and pathway names cached on 30 September 2026. Both analyses used minGSSize = 10, maxGSSize = 500, pAdjustMethod = “BH”, pvalueCutoff = 1, and qvalueCutoff = 1. All tested terms were retained, and BH-adjusted *p* < 0.05 defined significant enrichment. Hypergeometric *p* values and BH adjustments were numerically cross-checked.

Enrichment plots report the number of overlapping genes and BH-adjusted *p* values. Where few terms were significant, exploratory top-ranked terms were also displayed and distinguished from FDR-significant terms. Selected lipid- and lysosome-related terms were added from the same complete results without changing the significance threshold. Enrichment among downregulated or upregulated genes does not, by itself, demonstrate functional suppression or activation of an entire pathway.

### 2.7. Cell-Cell Communication Analysis

Cell communication was inferred using CellChat (v2.2.0.9001) and the accompanying CellChatDB.human database [15]. The input was the log-normalized RNA data layer, not integrated expression values. Excitatory neurons, inhibitory neurons, astrocytes, oligodendrocytes, oligodendrocyte precursor cells (OPCs), microglia, endothelial cells, and pericytes were included; Unknown nuclei were excluded. Nuclei were pooled by disease group to build separate Control and TSC networks. Secreted Signaling, ECM-Receptor, and Cell-Cell Contact interactions were retained.

Objects were constructed with createCellChat using the final cell-type labels. Following subsetData, overexpressed genes and interactions were identified using identifyOverExpressedGenes (do.fast = FALSE, thresh.pc = 0.1, thresh.fc = 0, thresh.*p* = 0.05) and identifyOverExpressedInteractions. The deduplicated union of candidate ligand-receptor pairs identified in either group was used for both networks. Communication probabilities were calculated with computeCommunProb (type = “triMean”, raw.use = TRUE, population.size = FALSE, nboot = 100, seed.use = 20261001), without protein-protein interaction projection. Cell types represented by fewer than 10 nuclei were filtered with filterCommunication. Pathway-level and aggregate networks were constructed using computeCommunProbPathway and aggregateNet (thresh = 0.05).

An interaction was considered detected if its within-network permutation *p* value was <0.05 and its inferred probability was positive. Interaction number denotes the number of detected ligand-receptor edges across the selected source-target combinations; interaction strength is the sum of their inferred probabilities. Group-pooled differences were treated as descriptive. Within-network permutation *p* values do not test differences between independent TSC and control donors.

### 2.8. snATAC-Seq Library Preparation, Processing, Quality Control, and Annotation

snATAC-seq libraries were prepared by Shanghai Applied Protein Technology Co., Ltd. using the 10× Genomics Chromium workflow described in the service report. Isolated nuclei were assessed and counted, then incubated with Tn5 transposase to fragment accessible DNA and introduce sequencing adapters. Transposed nuclei were partitioned with barcoded gel beads into GEMs. Linear amplification incorporated nucleus-specific barcodes; following emulsion breakage and product recovery, PCR introduced library adapters. Libraries were sequenced on an Illumina NovaSeq platform. The service report describes read-pair removal when either read contained more than three ambiguous bases or at least 20% of bases had quality scores below 5.

Four independent snATAC-seq libraries, comprising two TSC and two control donors from the snRNA-seq cohort, were analyzed. Cell Ranger ATAC (v2.1.0) generated fragment files and peak-barcode matrices using the refdata-cellranger-arc-GRCh38-2020-A-2.0.0 reference. Downstream analysis used Signac (v1.17.1) [22] and Seurat (v5.5.1) [18]. Initial chromatin objects required peaks detected in at least 10 nuclei and nuclei with at least 200 detected features. TSS enrichment and nucleosome signal were calculated with TSSEnrichment (fast = TRUE) and NucleosomeSignal. Nuclei were retained if fragments in peaks were >1000 and <25,000, the percentage of fragments in peaks was >10%, the blacklist ratio was <0.1, the nucleosome signal was <2, and TSS enrichment was >3. Doublets were identified separately per library using scDblFinder (v1.23.4; aggregateFeatures = TRUE, nfeatures = 25, clusters = FALSE), with mbkmeans (v1.22.0) feature aggregation. Only QC-passing singlets were retained. Per-donor QC and TSS score distributions are provided in Supplementary Figure S1. The principal ATAC atlas retained 25,314 nuclei, including 6040 annotated microglial nuclei. Two microglial nuclei had uncertain BAM/BAM-like transfer predictions (2/6040, 0.033%) and were retained in the primary analysis.

Chromatin counts underwent TF-IDF normalization, feature selection (FindTopFeatures, min.cutoff = “q5”), and singular value decomposition. Harmony (v2.0.5) adjusted the latent semantic indexing (LSI) representation for sample identity (dimensions 2–30, lambda = 0.8) [23]. The corrected dimensions 2–15 were used for UMAP, neighbor construction, and clustering (resolution = 1, algorithm = 3; seed 20261003). Gene-activity scores were used for RNA-assisted label transfer from the final snRNA-seq annotations, excluding Unknown reference nuclei. Canonical correlation analysis (CCA) transfer anchors and LSI-weighted transfer were used; assignments required a maximum transfer score ≥ 0.5 and a margin ≥ 0.15 over the second-highest score, otherwise nuclei were labeled Unknown. These scores are annotation support metrics, not calibrated probabilities.

### 2.9. Microglial Pseudobulk Construction and Differential Accessibility Analysis

Microglial nuclei were extracted after QC, doublet exclusion, and annotation. Fragment files were subset by donor-specific microglial barcodes to generate four donor-level pseudobulks. MACS2 (v2.2.9.1) was run separately for each donor with -f BEDPE -g hs --keep-dup all -q 0.01. Each fragment record was counted once, without expansion by duplicate-support counts. GenomicRanges (v1.58.0) was used to merge overlapping donor peaks. Consensus regions were required to have support from at least two donors, lie on autosomes, have lengths > 20 bp and <10 kb, and not overlap the hg38 blacklist supplied with Signac.

Signac FeatureMatrix generated nucleus-level counts for the consensus regions, which were summed within donors to construct a peak-by-donor count matrix. edgeR (v4.4.0) filterByExpr removed low-count peaks, and trimmed mean of M-values (TMM) normalization adjusted library composition. A negative binomial model was fitted with group as the explanatory variable.

Dispersion estimation and quasi-likelihood fitting used estimateDisp and glmQLFit with robust = TRUE, followed by glmQLFTest for the TSC-versus-Control comparison [24]. BH-FDR < 0.05 defined differentially accessible regions (DARs); no additional absolute log2 fold-change threshold was applied. Positive and negative log2 fold changes denote increased and decreased accessibility in TSC. DESeq2 (v1.46.0) was used for a sensitivity analysis on the same tested peaks, with a group-only design, independent filtering disabled, and automatic outlier replacement disabled. Normalized log counts per million (logCPM) values were used to inspect donor-level patterns; individual nuclei were not treated as independent biological replicates. The TSS3/4/6 sensitivity scenarios combined TSS enrichment thresholds of 3, 4, or 6 with FRiP thresholds of 10%, 15%, or 20%. All nine scenarios also used stricter nucleosome-signal and fragments-in-peaks thresholds (1.5 and 1500–20,000, respectively) than the primary analysis (2 and 1000–25,000). Comparisons with the primary analysis therefore assess sensitivity to combined filtering criteria, rather than changes in TSS enrichment or FRiP alone. Depth- and QC-sensitivity results are provided in Supplementary Figure S8 and Table S8. Method concordance was evaluated using Pearson and Spearman correlations of log2 fold changes, exact sign agreement across finite paired coefficients, and the overlap of BH-FDR-significant DAR sets. Five prespecified GREAT-linked candidate regions were compared individually. The same-universe method comparison and DAR counts across depth scenarios are shown in Supplementary Figure S9 and Table S8; donor-normalized candidate-region profiles are shown in Supplementary Figure S10. DEG_depth_sensitivity analysis results are shown in Supplementary Table S10.

### 2.10. GREAT Enrichment and Region-Gene Associations

Genomic region enrichment was performed locally using rGREAT (v2.8.0) [16]. Increased- and decreased-accessibility DARs were analyzed separately, using all 70,194 tested peaks as background. Regulatory domains were constructed from TxDb.Hsapiens.UCSC.hg38.knownGene (v3.20.0) with the basalPlusExt rule: a basal region from 5 kb upstream to 1 kb downstream of each transcription start site (TSS), extended toward neighboring basal domains up to 1 Mb.

GO Biological Process annotations were obtained from org.Hs.eg.db and GO.db (both v3.20.0). Local rGREAT region-level binomial tests were BH-adjusted, with FDR < 0.05 defining significant enrichment. Predicted region-gene associations were intersected with significant microglial RNA DEGs. In the displayed RNA-DAR quadrant plot, each point represents a predicted link between a significant DAR and a significant RNA DEG. The *x*-axis is the edgeR DAR log2 fold change, and the *y*-axis is the DESeq2 RNA log2 fold change. Gene counts are deduplicated within each quadrant; a gene linked to DARs with opposite directions can occur in more than one quadrant. These proximity-based associations are predictions, not experimentally established enhancer-target links.

### 2.11. Motif Footprinting

Motif footprinting used Signac (v1.17.1). The reported profiles were generated from the 6040 microglial nuclei retained in the primary ATAC analysis. Human motifs for SPI1, CEBPB, TFEB, TFE3, and PPARG were obtained from JASPAR2020 (R package v0.99.10) using TFBSTools (v1.44.0), retaining the latest available version of each motif within that release. motifmatchr (v1.28.0) identified matches in the shared peak set using BSgenome.Hsapiens.UCSC.hg38 (v1.4.5) and *p*.cutoff = 5 × 10^−5^. Duplicate matches and sites near chromosome boundaries were removed; motifs were required to have at least 100 sites on autosomes or sex chromosomes. T additional PPARG profile is provided in Supplementary Figure S11 as a descriptive analysis.

Donor-specific Tn5 hexamer bias was estimated from chromosome 1 using InsertionBias. Footprint profiles were calculated at fixed shared motif sites over ±250 bp (compute.expected = TRUE, in.peaks = TRUE). After normalization, expected signal was subtracted from observed signal to generate bias-corrected curves. Group curves were the equal-weight mean of the two donor curves. Descriptive footprint depth was calculated as mean flanking signal, 10–50 bp beyond motif boundaries, minus mean motif-center signal. Leave-one-donor-out checks assessed direction stability. Footprints were interpreted descriptively, without nucleus-level group tests or claims of directly measured transcription factor occupancy. TFEB/TFE3 donor-omission profiles are provided in Supplementary Figure S12 and Table S9.

### 2.12. chromVAR Analysis

Motif-associated accessibility was quantified using chromVAR (v1.28.0) [17] on the microglial peak-by-nucleus count matrix and human JASPAR2020 CORE motif matches. GC bias was estimated with addGCBias. Fifty background peak sets were generated with getBackgroundPeaks (seed 20261004), and bias-corrected deviations were calculated with computeDeviations. Deviation z-scores were averaged within each donor; group means were assigned equal weight to donors. Motifs were ranked by the absolute difference between the TSC and Control donor means, and the top 50 were displayed. Scores were not rescaled within rows, and selection did not use a motif-level significance threshold. Motif scores were not interpreted as direct measurements of transcription factor activity. Candidate motif donor-omission summaries are provided in Supplementary Figure S13 and Table S9.

### 2.13. Statistical Analysis

The independent donor was the biological replicate for disease-associated differential expression (three donors per group) and differential accessibility (two donors per group). The primary tests were the DESeq2 negative binomial Wald test for RNA and the edgeR quasi-likelihood F-test for accessibility. BH correction was applied separately within each tested cell-type gene set, the tested peak set, and each directional enrichment analysis within each database. Significance thresholds and effect-size filters are specified in the corresponding sections.

Cell-type proportions calculated from pooled nuclei, CellChat group-pooled networks, donor-averaged chromVAR scores, and footprint curves were descriptive analyses. Cell-marker selection was an annotation-support analysis and was not used as a donor-level disease test. With only three RNA donors and two ATAC donors per group, covariate-adjusted effects could not be estimated reliably. Dispersion sharing and robust estimation stabilize small-sample models, and BH correction addresses multiplicity; neither increases the number of independent donors nor removes confounding.

Primary computational analyses used R v4.4.3 and the package versions specified above. Supplementary QC plots and the Top50 chromVAR heatmap were rendered in R v4.5.3 without recalculating the primary statistical results. The reported FDRs should be interpreted in the context of the small, exploratory cohort.

## 3. Results

### 3.1. Characteristics of Patients

The RNA cohort comprised three children with TSC-associated epilepsy and three pediatric surgical controls: two with post-traumatic epilepsy (CONTROL2 and CONTROL3) and one with cerebral cavernous malformation without seizures (CONTROL1). The ATAC subset comprised PATIENT1, PATIENT3, CONTROL1, and CONTROL2. All participants were minors, and written informed consent was obtained from their legal guardians. Donor characteristics are summarized in Supplementary Table S1.

### 3.2. Single-Nucleus Transcriptomic Profiling and Quality Control

After quality control and doublet removal, 50,148 RNA nuclei remained. Subsequent exclusion of 109 supported or uncertain BAM-like candidates left 50,039 nuclei for the current atlas: 29,982 from TSC donors and 20,057 from controls. The microglial subset contained 6520 nuclei (3897 TSC and 2623 Control). Donor-level nucleus counts and QC metrics are provided in Supplementary Table S2. QC distributions and pseudobulk ordination by PCA and multidimensional scaling (MDS) are shown in Supplementary Figure S2.

### 3.3. Cell Type Identification

**Figure 1:**
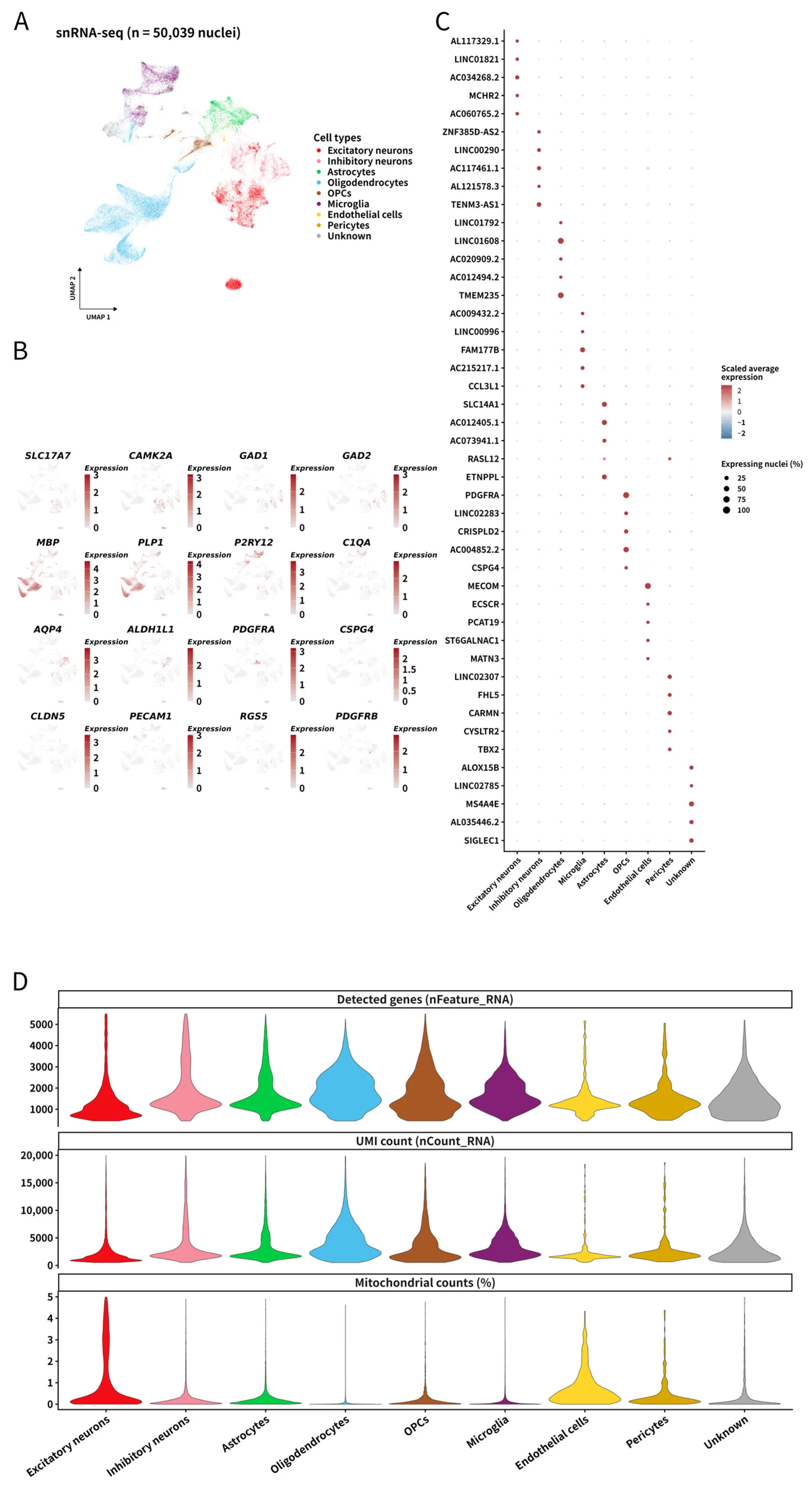
Single-nucleus transcriptomic atlas of human brain tissue from tuberous sclerosis complex (TSC) and control samples. (**A**) Uniform manifold approximation and projection (UMAP) of 50,039 retained nuclei from three TSC donors (29,982 nuclei) and three control donors (20,057 nuclei), colored by final annotation. Eight named cell types and the unresolved Unknown population are displayed. (**B**) Feature plots of canonical markers. Color denotes log-normalized RNA expression, with the upper display limit set to the gene-specific 99th percentile; gray indicates low expression. (**C**) Dot plot of the selected top-five positive markers per population. Dot area denotes the percentage of nuclei with detectable expression; color denotes mean expression scaled by gene across populations. Marker selection is distinct from TSC-versus-Control differential testing. (**D**) Per-cell-type quality control (QC) distributions for detected genes (nFeature_RNA), UMI counts (nCount_RNA), and mitochondrial transcript percentage (percent.mt). These are nucleus-level descriptive distributions, not independent donor replicates.

Clustering and marker-based annotation resolved eight named cell types and an unresolved population labeled Unknown (Figure 1A). Canonical markers supported the annotations of excitatory neurons, inhibitory neurons, astrocytes, endothelial cells, microglia, OPCs, oligodendrocytes, and pericytes (Figure 1B). The top five positive markers selected for each annotated population are summarized in Figure 1C. Distributions of detected genes, UMI counts, and mitochondrial count percentages are shown by cell type in Figure 1D.

### 3.4. Cell Composition and Cell-Type-Specific Differential Expression

**Figure 2:**
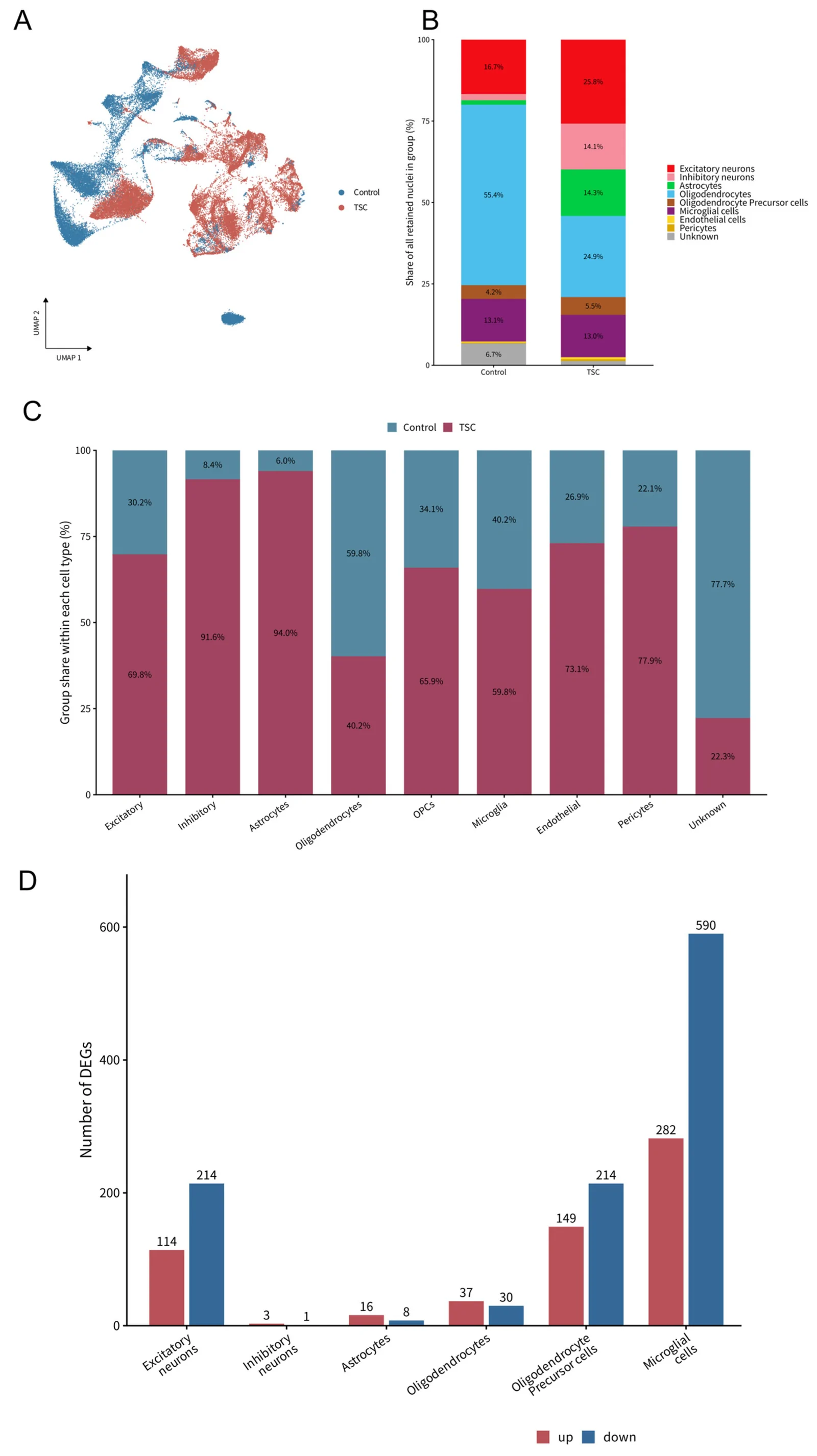
Descriptive cell composition and donor-level differential expression. (**A**) UMAP colored by group (Control and TSC). (**B**) Cell-type composition within each group; the denominator is all retained nuclei in that group. (**C**) Contribution of TSC and Control to each cell type; the denominator is all nuclei of that type across both groups. Both composition panels pool nuclei across three donors per group. (**D**) Numbers of upregulated and downregulated differentially expressed genes (DEGs) in six cell types, using donor-level DESeq2 Benjamini–Hochberg false discovery rate (BH-FDR) < 0.05 and |DESeq2 model log2FC| ≥ 0.25.

The pooled distribution of retained nuclei differed between groups (Figure 2A–C). Within-group proportions were higher in TSC for excitatory neurons (25.8% versus 16.7% in controls) and lower for oligodendrocytes (24.9% versus 55.4%). Microglia accounted for similar pooled proportions (13.0% versus 13.1%). These percentages describe nuclei recovered by sequencing, not absolute tissue cell abundance. Figure 2C uses a different denominator: it shows each group’s contribution to the total nuclei of a given cell type. Neither pooled display constitutes a donor-level test of differential abundance.

Among the six cell types displayed for donor-level differential expression, microglia had the largest DEG set, with 282 upregulated and 590 downregulated genes in TSC (Figure 2D). DEG counts depend on statistical power and do not directly rank the biological importance of cell types. The OPC comparison met the coverage criterion of at least 10 nuclei in each of all six donors. Endothelial cells, pericytes, and Unknown nuclei did not meet this criterion. Results are provided in Supplementary Table S3.

### 3.5. Downregulated Microglial Genes Involve Lipid Transport and Endosomal-Lysosomal Processes

**Figure 3:**
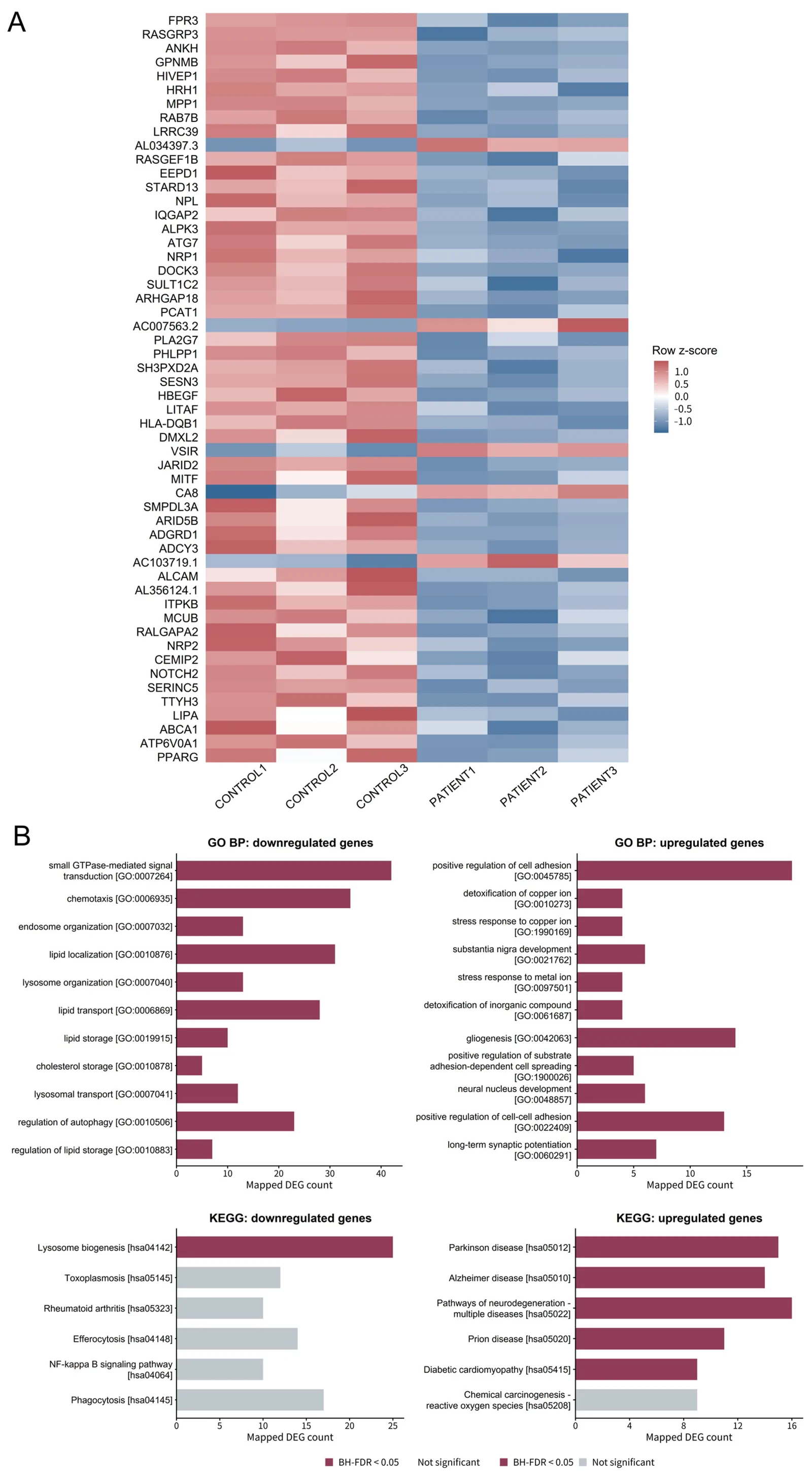
Microglial differential expression and functional enrichment. (**A**) Donor-level heatmap of selected microglial genes, including LIPA, ABCA1, ATP6V0A1, and GPNMB. Each column represents one donor (three per group). Colors are row-wise z-scores of log2(DESeq2 size-factor-normalized pseudobulk counts + 1), not *p* values. Genes were selected by differential-expression ranking and biological relevance. (**B**) Gene Ontology (GO) Biological Process and Kyoto Encyclopedia of Genes and Genomes (KEGG) ORA of upregulated and downregulated DEGs. Bar length represents overlapping gene count; burgundy denotes BH-FDR < 0.05 and gray denotes non-significant exploratory terms. Selected lipid- and lysosome-related terms were added from the same tested results. RNA DEGs met BH-FDR < 0.05 and |DESeq2 model log2FC| ≥ 0.25; enrichment used the corresponding tested, mapped gene background.

The strict microglial analysis identified 872 DEGs, comprising 282 upregulated and 590 downregulated genes. ABCA1, LIPA, ATP6V0A1, GPNMB, and PPARG were all significantly lower in TSC (Figure 3A). Directional ORA of downregulated genes identified lipid transport, lysosome organization, lipid storage, and cholesterol storage, together with KEGG hsa04142 (Figure 3B). The five candidates retained significant negative effects in all six RNA leave-one-donor-out analyses. Enrichment for lysosome organization, lipid storage, and cholesterol storage was significant in all six omission runs, whereas hsa04142 was significant in three. These findings support a reproducible gene-level direction and selected enrichment themes, not uniformly donor-insensitive pathway significance or demonstrated impairment of lipid flux, acidification, or degradation. Candidate expression and omission estimates are shown in Supplementary Figure S3. DEG-set and pathway retention are shown in Supplementary Figure S4 and detailed in Supplementary Table S5; complete ORA results are provided in Supplementary Table S4. Consistent with the directional ORA results, preranked GSEA identified negative enrichment of the KEGG lysosome-related gene set hsa04142 (NES = −2.18; BH-FDR = 8.79 × 10^−11^), GO lysosome organization (GO:0007040; NES = −2.02; BH-FDR = 1.06 × 10^−4^), lipid transport (GO:0006869; NES = −1.63; BH-FDR = 3.18 × 10^−4^), and lipid storage (GO:0019915; NES = −1.99; BH-FDR = 3.86 × 10^−4^; Supplementary Figure S6; Table S7).

### 3.6. Inferred Microglial Communication Differs Between Groups

**Figure 4:**
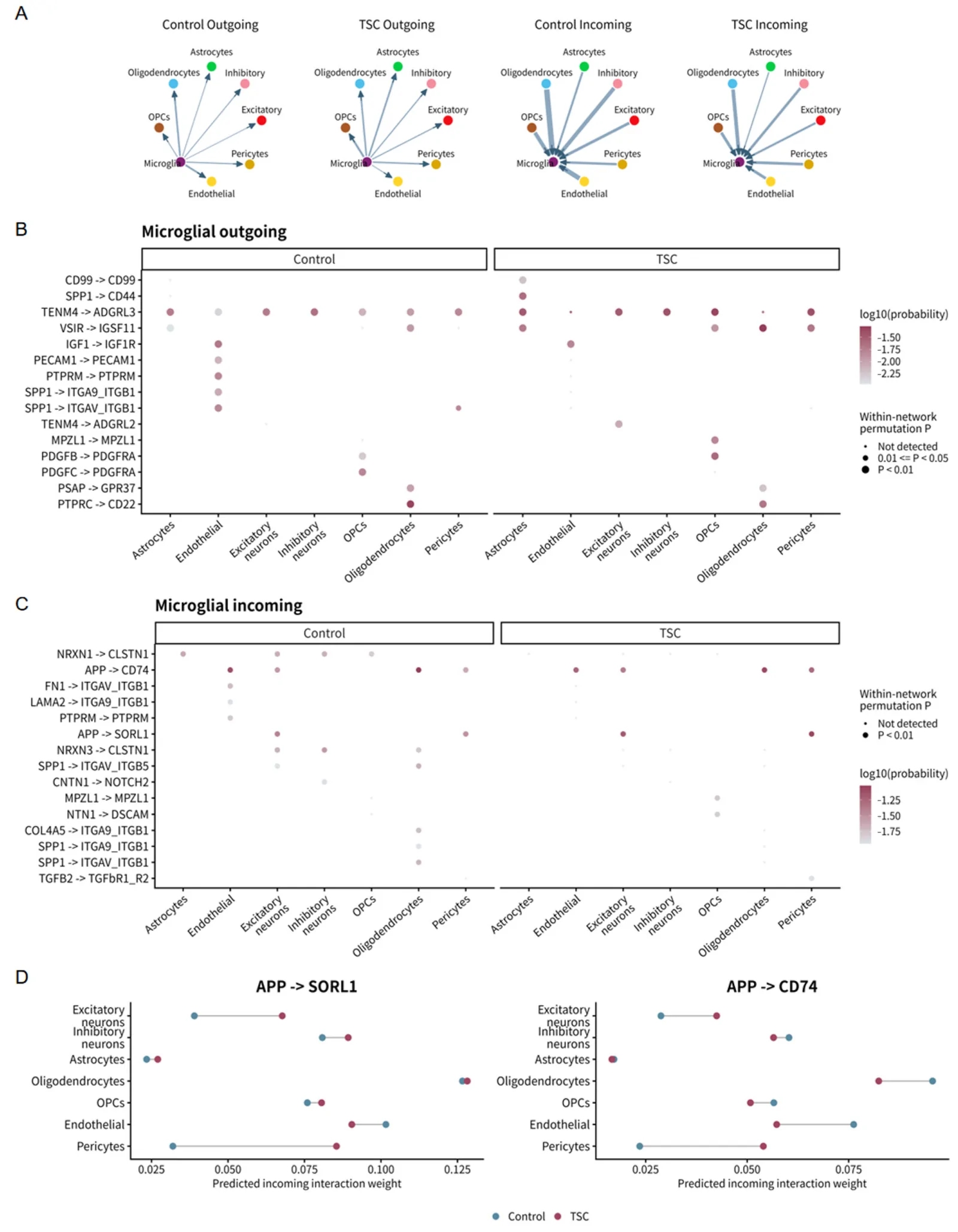
Inferred communication involving microglia. (**A**) Group-pooled network diagrams for outgoing and incoming microglial interactions. Nodes identify cell types and have fixed sizes; arrow direction denotes source-to-target signaling, and edge width represents the summed inferred interaction weight on a common scale. (**B**,**C**) Selected outgoing (**B**) and incoming (**C**) ligand-receptor pairs. Rows identify pairs; columns identify partner cell types within each group. Color denotes log10(inferred communication probability). Dot size encodes within-group permutation *p*-value categories (large, *p* < 0.01; small, 0.01 ≤ *p* < 0.05); tiny pale marks indicate interactions not meeting the detection criterion. Dot size does not represent expression or communication probability. (**D**) Inferred APP-SORL1 and APP-CD74 input from each indicated source population to microglia, shown for Control and TSC. Lines connect group-pooled estimates, not paired donor measurements. None of these within-network *p* values is a test of the difference between disease groups.

CellChat inferred incoming and outgoing microglial interactions with the other annotated major cell types in both groups (Figure 4A). Individual ligand-receptor pairs showed source- and target-dependent patterns rather than a uniform change (Figure 4B,C). APP- CD74 input to microglia had higher inferred weights in TSC for most cell types, whereas APP-SORL1 changes varied by source (Figure 4D).

### 3.7. A Complementary snATAC-Seq Cell Atlas

**Figure 5:**
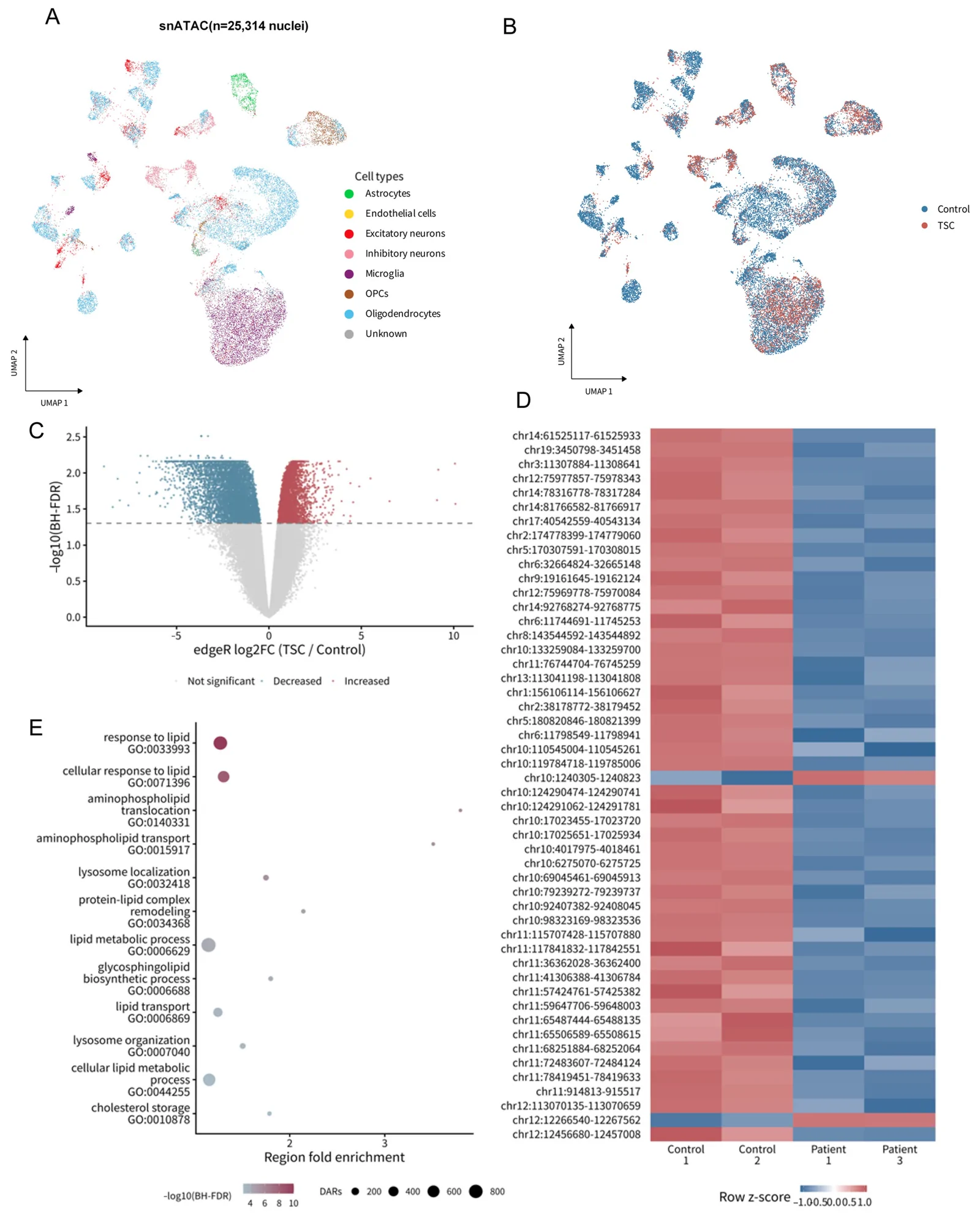
Microglial chromatin accessibility and predicted functional associations. (**A**,**B**) UMAP of 25,314 retained single-nucleus assay for transposase-accessible chromatin using sequencing (snATAC-seq) nuclei from two Control and two TSC donors, colored by RNA-assisted cell-type annotation (**A**) or group (**B**). (**C**) Volcano plot of donor-pseudobulk edgeR quasi-likelihood results for 70,194 tested peaks. The *x*-axis shows log2 fold change (TSC/Control); the *y*-axis shows −log10(BH-FDR). The dashed line marks FDR = 0.05. Increased- and decreased-accessibility differentially accessible regions (DARs) are indicated in red and blue, respectively (3443 and 5840 peaks). (**D**) The 50 DARs with the lowest FDR (ties ordered by decreasing absolute log2 fold change) across the four donors, displayed as row-wise z-scores of normalized log counts per million (logCPM). (**E**) Selected GO Biological Process terms from local GREAT analysis of decreased-accessibility DARs, using all tested peaks as background. The *x*-axis denotes region fold enrichment, point size denotes the number of overlapping DA peaks, and color denotes −log10(BH-FDR).

QC and doublet exclusion retained 25,314 snATAC-seq nuclei from two TSC and two control donors. RNA-assisted annotation identified seven named cell types and an Unknown population (Figure 5A,B). A separately resolved pericyte population was not represented in this annotation. Donor-level QC metrics and TSS enrichment distributions are shown in Supplementary Figure S1.

### 3.8. Differential Accessibility Is Associated with Lipid- and Lysosome-Related Processes

Microglial accessibility was analyzed using four donor pseudobulks, comprising 1146 and 2070 nuclei from the two control donors and 1085 and 1739 nuclei from the two TSC donors. Of 70,194 tested peaks, 9283 met BH-FDR < 0.05: 3443 had increased, and 5840 had decreased accessibility in TSC (Figure 5C). A heatmap summarizes selected DARs across donors (Figure 5D). These exploratory estimates are based on two independent donors per group. In the equal-depth sensitivity analysis, each donor contributed 17,383,212 unique microglial fragment records. Rebuilding the consensus with support from at least two donors yielded 57,883 tested regions, of which 8791 were differentially accessible (3315 increased and 5476 decreased; Supplementary Table S8). Across the same 70,194 peaks, edgeR and DESeq2 log2 fold changes were strongly correlated (Pearson r = 0.9951; Spearman rho = 0.9934), with 97.27% sign agreement and a median absolute coefficient difference of 0.0346. DESeq2 identified 9994 DARs (3721 increased and 6273 decreased), of which 8598 overlapped the 9283 edgeR DARs (Jaccard index = 0.8051). All five GREAT-linked regions associated with ABCA1 (two), ATP6V0A1 (one), and GPNMB (two) had negative effects and BH-FDR < 0.05 in both models; they also retained significance in both models after equal-depth thinning with the original consensus held fixed. This agreement supports robustness to model choice within the same cohort, not independent replication (Supplementary Figure S9; Table S8).

GREAT analysis of decreased-accessibility DARs, relative to the tested peak background, identified GO Biological Process terms including lipid transport (GO:0006869; BH-FDR = 4.12 × 10^−4^), lysosome organization (GO:0007040; BH-FDR = 4.45 × 10^−4^), and lysosome localization (GO:0032418; BH-FDR = 4.37 × 10^−6^; Figure 5E). This regional enrichment echoes the lipid and endosomal-lysosomal themes in the RNA analysis, without implying identical enriched gene sets or direct functional validation. Using this rebuilt consensus, decreased-accessibility regions remained enriched for lysosome localization (BH-FDR = 1.02 × 10^−6^), lipid transport (BH-FDR = 2.79 × 10^−4^), and lysosome organization (BH-FDR = 1.85 × 10^−3^). Depth- and QC-sensitivity displays are provided in Supplementary Figure S8, with the scenario-specific results and nucleus counts in Supplementary Table S8.

GREAT linked ABCA1, ATP6V0A1, and GPNMB to two, one, and two decreased-accessibility DARs, respectively. At these regions, normalized signals were lower in both TSC donors than in both controls (Supplementary Figure S10; Table S9), concordant with the RNA direction. None of these five peaks lies within TSS ±2 kb under the gene annotation used here. They therefore provide predicted distal region-gene associations, not evidence of promoter closure or experimentally validated enhancers. LIPA was downregulated in RNA but was not linked to a significant DAR in this analysis.

### 3.9. Cross-Omic Directional Concordance and Motif-Associated Accessibility

**Figure 6:**
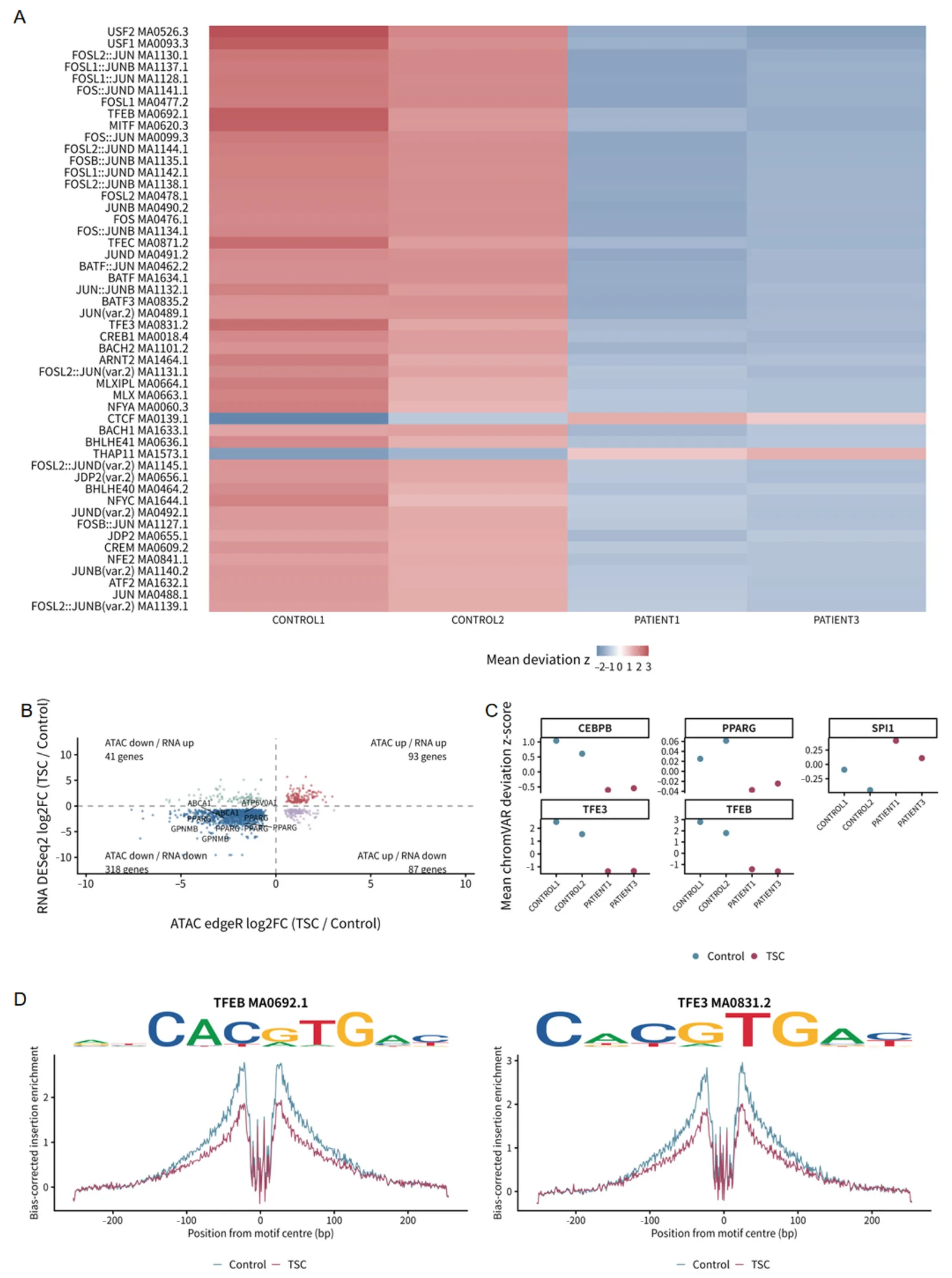
Exploratory cross-omic and motif analyses in microglia. (**A**) Donor-averaged chromatin variation across regions (chromVAR) deviation z-scores for the 50 motifs with the largest absolute TSC-minus-Control mean differences. TFEB and TFE3 are included by this ranking. Scores are not row-standardized, and ranking is not a significance test. (**B**) Each point is a GREAT-predicted link between a significant differentially accessible region (DAR) and an RNA DEG. The *x*-axis shows edgeR DAR log2FC and the *y*-axis DESeq2 RNA log2FC. Counts denote unique genes within each quadrant: 93 concordant increases, 318 concordant decreases, 41 RNA-up/ATAC-down, and 87 RNA-down/ATAC-up. Across the plot, there are 458 unique genes; 81 occur in multiple quadrants. Dashed lines indicate zero. (**C**) Donor-averaged chromVAR scores for CEBPB, PPARG, SPI1, TFE3, and TFEB. Each point represents one independent donor (two per group). Blue and burgundy denote Control and TSC. These distributions do not establish donor-level significance. (**D**) JASPAR sequence logos and normalized, Tn5-bias-corrected footprint profiles for TFEB MA0692.1 and TFE3 MA0831.2. Curves are equal-weight means of two donor profiles over ±250 bp. Logos depict motif preferences, not sequences at a single candidate-gene locus; profiles do not directly establish factor-specific occupancy.

The chromVAR heatmap displays the 50 motifs with the largest absolute differences between group-mean donor scores (Figure 6A). TFEB and TFE3 were included without forced addition. Their scores were lower in both TSC donors than in both controls. This ranking describes the magnitude of observed differences and does not identify statistically significant motifs.

Intersecting the 872 microglial RNA DEGs with significant GREAT-linked DARs yielded 458 unique genes. The link-level quadrant plot contained 93 genes with increased RNA and increased accessibility and 318 with decreased RNA and decreased accessibility; the discordant quadrants contained 41 genes with increased RNA/decreased accessibility and 87 with decreased RNA/increased accessibility (Figure 6B). These are unique-gene counts within each quadrant, not mutually exclusive gene totals: 81 genes occurred in more than one quadrant because their linked DARs had different directions. Selected ABCA1, ATP6V0A1, and GPNMB associations showed concordant decreases.

The candidate-level plots showed lower donor-averaged scores for CEBPB, PPARG, TFE3, and TFEB in both TSC donors than in either control (Figure 6C). SPI1 showed the opposite pattern. PPARG exhibited a smaller absolute difference than the other displayed motifs. These donor-level distributions describe motif-associated accessibility, not statistically established differences in transcription factor expression, binding, or activity.

Bias-corrected footprint profiles, displayed with their corresponding motif sequence logos, showed different insertion patterns around TFEB and TFE3 sites in the two groups (Figure 6D). Together with chromVAR, these profiles indicate differences in motif-associated chromatin features. Additional CEBPB, PPARG, and SPI1 profiles and TFEB/TFE3 omission analyses are shown in Supplementary Figures S11 and S12, respectively.

Overall, selected downregulated lipid- and lysosome-related genes were accompanied by decreased accessibility at predicted associated regions and by changes in TFEB/TFE3-associated motif features. These observations support a testable regulatory hypothesis.

## 4. Discussion

After excluding 109 supported or uncertain BAM-like RNA nuclei, this exploratory study identified 872 donor-level microglial DEGs, with downregulated genes enriched for lipid-handling and endosomal-lysosomal annotations. The strongest evidence concerns the direction of selected candidate-gene changes, which persisted across donor-omission analyses. Chromatin profiles supplied complementary evidence at predicted regions associated with ABCA1, ATP6V0A1, and GPNMB. Together, these observations nominate post-engulfment processing and lipid export for investigation.

The candidate pattern spans intracellular processing and export rather than a single transporter. LIPA and ABCA1 suggest that processing lipid cargo and redistributing its products could both be altered, while ATP6V0A1 and GPNMB nominate related endolysosomal functions. This interpretation is consistent with enrichment among downregulated genes, but only some enrichment terms survived every donor omission. ABCA1 is a useful cross-omic candidate because its transcript level and accessibility at two GREAT-predicted associated regions were lower in TSC. Experimental ABCA1 deficiency reduces glial cholesterol efflux to apolipoproteins [25], providing a functional rationale for testing export. Our observations, however, do not demonstrate altered ABCA1 protein, cholesterol accumulation, or reduced efflux. LIPA downregulation points to lysosomal lipid-cargo processing upstream of export. Lysosomal cholesteryl ester hydrolysis supports repair-associated microglial states in experimental white-matter injury [26], while chronically challenged TREM2-deficient microglia can internalize myelin yet fail to clear its cholesterol efficiently [27]. These studies motivate distinguishing uptake from subsequent processing in our tissue. They do not establish the same mechanism in TSC. LIPA had no linked significant DAR, so its support here remains transcriptional rather than concordant across both assays. The ATP6V0A1 and GPNMB results extend the candidate signature to post-engulfment processing. Experimental evidence links v0-ATPase a1 to microglial phagosomal fusion [28], and defective cholesterol clearance can damage phagolysosomes after myelin injury [29]. GPNMB warrants particular caution: seizure-related experimental work reported its induction and a protective phagocytic role, whereas its RNA was lower in our TSC samples [30]. That study implicated ATP6V1A, not ATP6V0A1, as its interaction partner. Disease stage, brain region, treatment, and the non-healthy controls are possible explanations for the different direction. PPARG downregulation provides an additional candidate transcriptional change; neither it nor the small chromVAR score difference proves a shared causal regulatory axis.

Together, the results support testing a potential imbalance between lipid-cargo processing and export in TSC-associated microglia. They do not establish a linear LIPA-ABCA1 pathway, lipid accumulation, failed acidification, or a causal link to epilepsy. Joint measurements of lysosomal lipase activity, cholesterol efflux, lipid burden, and phagosome maturation would resolve where any functional limitation occurs. Demonstrating an effect on neuronal network excitability would then be necessary to connect that limitation to epileptogenesis.

The inferred communication results place these intracellular changes in a broader cellular context. APP-CD74 input was higher for most source populations, whereas APP-SORL1 changes depended on the source. Experimental work implicates SORLA in lysosomal sorting of amyloid-β [31] and shows that CD74 can interact with APP and alter its processing [32]. These observations motivate investigation of cargo handling, but CellChat estimates from RNA do not measure receptor localization, substrate uptake, or degradation. Increased interaction weights could reflect altered expression or a compensatory response; they do not demonstrate amyloid deposition or altered flux.

The chromatin results strengthen the rationale for studying selected candidates without proving their regulation. ABCA1, ATP6V0A1, and GPNMB were downregulated and linked by GREAT to decreased-accessibility regions outside TSS ±2 kb. Such distal associations are compatible with regulatory involvement but remain proximity-based predictions. As the assays came from the same cohort and used separate nuclei, agreement between them is complementary evidence rather than independent replication or a within-cell molecular correlation.

Lower TFEB- and TFE3-associated chromVAR scores nominate lysosomal regulatory programs for follow-up. TFEB and TFE3 can regulate lysosomal and autophagy-related genes in experimental systems [33,34]. Nevertheless, similar DNA-binding motifs can be shared among family members, and neither chromVAR nor footprinting directly measures factor-specific activity. The observed accessibility pattern therefore does not establish that TFEB or TFE3 caused the RNA changes. Local accessibility, sequencing depth, residual sequence bias, and motif similarity can influence the profiles; they do not establish factor-specific occupancy or regulation of the nominated genes. With only two donors per group, the DESeq2 analysis is presented as a descriptive sensitivity check. Depth/QC sensitivity and motif donor-omission results are shown in Supplementary Figures S8, S12 and S13 and Tables S8 and S9.

The TSC-mTOR context is relevant but does not justify a simple causal chain. TFEB regulation involves substrate-specific nutrient-sensing mechanisms that differ from canonical growth-factor-RHEB signaling [35]. Consequently, TSC-associated mTORC1 dysregulation does not by itself predict the direction of TFEB/TFE3 activity in these human microglia. Protein localization, phosphorylation, locus-specific occupancy, and perturbation or rescue experiments are needed to test the proposed connection.

The principal limitation is the small number of independent donors: three per group for RNA and two per group for ATAC. Pseudobulk analysis avoids nucleus-level pseudoreplication, but statistical moderation cannot compensate for limited replication or remove clinical confounding. Potential influences include age, brain region, medication, seizure burden, and tissue handling; QC metrics also differed across ATAC donors. Obtaining human brain tissue, particularly healthy pediatric tissue, is difficult. Consequently, controls were children undergoing surgery for post-traumatic epilepsy and pediatric cerebral cavernous malformation without seizures, not fully healthy brain-tissue controls. Brain tissue from CONTROL2 and CONTROL3 was obtained during second operations for epilepsy, performed 15 and 13 months after their initial traumatic brain injury surgeries, respectively. Although these intervals place tissue collection beyond the acute injury period, an influence of chronic-phase microglial states cannot be fully excluded. This control selection limits extrapolation to differences between TSC lesions and healthy pediatric brain tissue. Individual-control candidate contrasts are shown in Supplementary Figure S14.

RNA-assisted ATAC annotation is not independent validation of cell identity, and predicted region-gene associations require functional confirmation. Group-pooled communication networks and motif summaries lack donor-level evidence of significant between-group differences. Cross-sectional resected tissue also cannot establish whether the molecular changes preceded seizures. We plan to expand this work through larger, multicenter studies with improved control sampling and more detailed clinical matching. Ethically available non-TSC tissues with better-characterized injury status, alongside suitable external reference datasets, may help distinguish disease-associated changes from control-related effects. Conservative removal of BAM-like candidates reduces one potential source of mixture but does not validate every retained identity or fully remove state-composition effects. Some exploratory states lacked coverage across all donors, and state assignments changed with UMI thinning. Depth and QC sensitivities likewise did not preserve every GREAT term under every setting. These limitations qualify, rather than negate, the candidate-level observations. Microglial identity results are provided in Supplementary Figure S7. State-coverage and RNA-depth sensitivity results are provided in Supplementary Figures S5 and S15 and Tables S6 and S10.

The next step is to validate LIPA, ABCA1, ATP6V0A1, and GPNMB expression and localization in independent tissue, followed by functional assays in an appropriate microglial model. TFEB/TFE3-dependent regulation would require perturbation, protein-localization measurements, and locus-specific evidence. In future studies, RNA in situ hybridization or protein staining will need to be added to validate the conclusions of this study. Rescue experiments linked to neuronal network recordings would be needed before assigning these candidates a causal role in epileptogenesis.

## 5. Conclusions

Donor-level snRNA-seq analysis suggests microglial remodeling involving lipid transport and endosomal-lysosomal processes in TSC-associated epileptogenic lesions. Decreased accessibility at predicted regions linked to ABCA1, ATP6V0A1, and GPNMB, together with TFEB/TFE3-associated motif changes, provides complementary regulatory leads. These findings provide a molecular basis for further investigation of the relationship between microglia and epileptogenesis, although they remain exploratory and warrant independent replication and functional validation before causal or therapeutic conclusions can be drawn.

## Data Availability

The minimally processed data matrices presented in the study and the analytical code are openly available in Figshare at DOI: 10.6084/m9.figshare.34783665. The raw sequencing data are currently available from the corresponding author upon reasonable request. These data will be deposited in the Genome Sequence Archive for Human (GSA-Human) at the National Genomics Data Center upon completion of the ongoing follow-up study.

## Supplementary Materials

The following supporting information can be downloaded at: https://www.mdpi.com/article/10.3390/biology15211976/s1. Supplementary Figure S1: Donor-level quality control of snATAC-seq libraries; Supplementary Figure S2: RNA donor quality control and pseudobulk ordination; Supplementary Figure S3: Candidate RNA expression and donor-omission sensitivity; Supplementary Figure S4: Gene-set and pathway-level donor-omission sensitivity; Supplementary Figure S5: Exploratory microglial state coverage and sensitivity to UMI thinning; Supplementary Figure S6: Preranked gene set enrichment analysis (GSEA) of microglial donor-level RNA differences; Supplementary Figure S7: Marker-based reassessment of microglial identity; Supplementary Figure S8: GREAT and motif enrichment sensitivity to sequencing depth and quality-control filtering; Supplementary Figure S9: Concordance of microglial assay for ATAC differential-accessibility models; Supplementary Figure S10: Donor accessibility at five candidate-associated regions; Supplementary Figure S11: Additional motif sequence logos and footprint profiles; Supplementary Figure S12: TFEB and TFE3 footprint donor-omission sensitivity; Supplementary Figure S13: TFEB and TFE3 chromVAR donor-omission sensitivity; Supplementary Figure S14: Candidate expression across individual surgical controls; Supplementary Figure S15: RNA sequencing depth and differential-expression sensitivity; Supplementary Table S1: Clinical characteristics of the study samples; Supplementary Table S2: RNA donor-level quality control and nucleus exclusion counts; Supplementary Table S3: RNA differential-expression results and cell-type donor coverage; Supplementary Table S4: Directional GO and KEGG over-representation analyses; Supplementary Table S5: RNA donor-omission sensitivity analyses; Supplementary Table S6: Exploratory microglial state coverage and state-restricted and state-balanced differential-expression analyses; Supplementary Table S7: Complete preranked GSEA results; Supplementary Table S8: ATAC sequencing-depth, quality-control filtering, and statistical-model sensitivity analyses; Supplementary Table S9: Candidate-associated regions, chromVAR scores, and footprint summaries; Supplementary Table S10: RNA sequencing-depth sensitivity analyses.
