# Supplementary material for: Exploratory Single-Nucleus Transcriptomic and Chromatin Accessibility Profiling Indicates Microglial Remodeling in Tuberous Sclerosis Complex-Associated Epilepsy
Source: Biology (Basel). 2026 Nov 2;15(21):1976. doi: 10.3390/biology15211976 (PMC13762191; doi:10.3390/biology15211976)
Supplement: Supplementary file 1 [file biology-15-01976-s001.zip › Supplementary Figures/Supplementary Figure S1.pdf]

Supplementary Figure S1

Donor-level quality control of snATAC-seq libraries

A

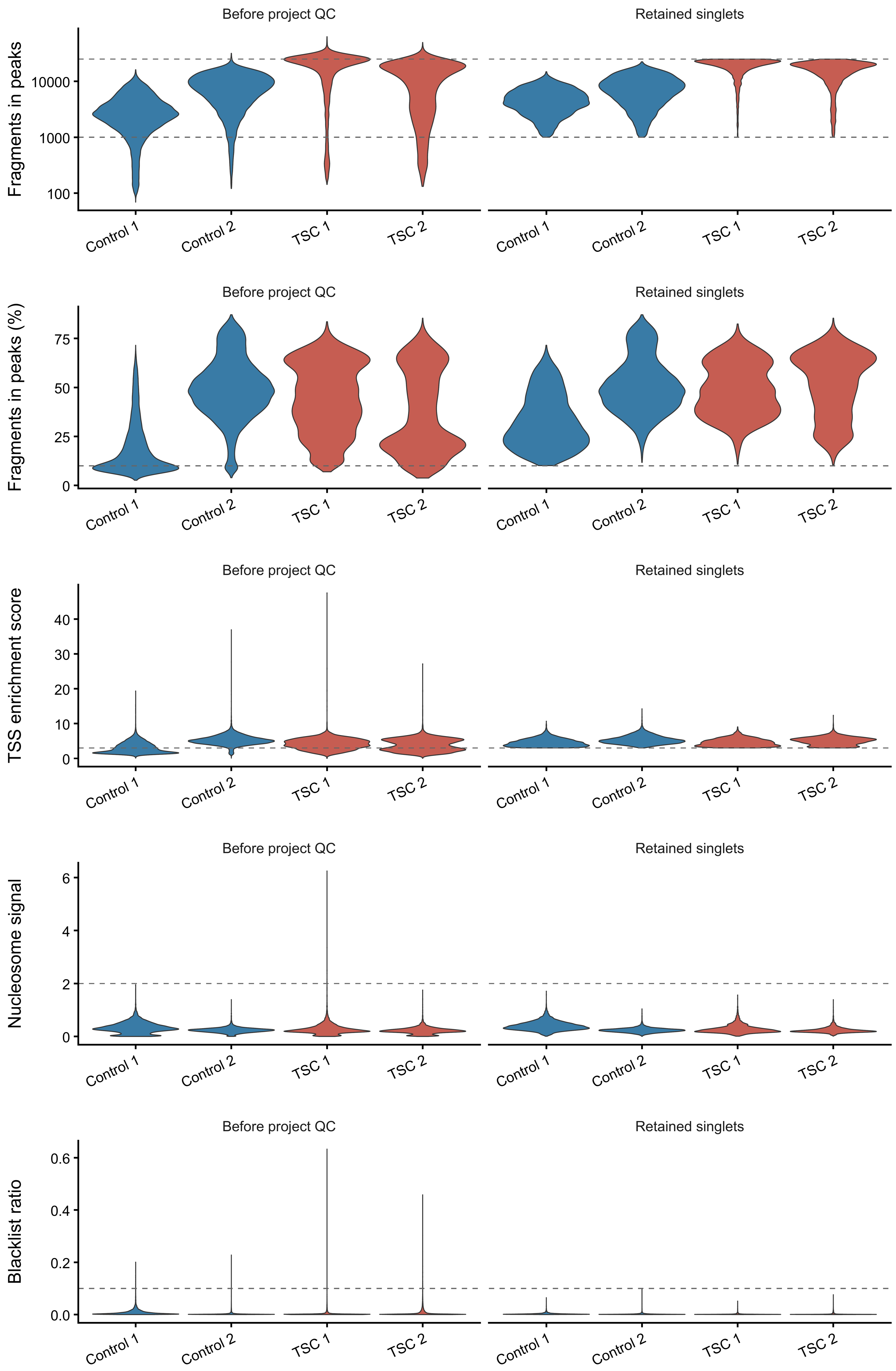

Figure legend

(A) Distributions of fragments in peaks, percentage of fragments in peaks, TSS enrichment, nucleosome signal, and blacklist ratio before project QC and after singlet selection. This archived all-cell QC display is reproduced without recalculation. Control 1 and Control 2 denote CONTROL1 and CONTROL2; legacy TSC 1 and TSC 2 denote PATIENT1 and PATIENT3. Distributions are descriptive nucleus-level summaries, not donor-level hypothesis tests.
