# Supplementary material for: Exploratory Single-Nucleus Transcriptomic and Chromatin Accessibility Profiling Indicates Microglial Remodeling in Tuberous Sclerosis Complex-Associated Epilepsy
Source: Biology (Basel). 2026 Nov 2;15(21):1976. doi: 10.3390/biology15211976 (PMC13762191; doi:10.3390/biology15211976)
Supplement: Supplementary file 1 [file biology-15-01976-s001.zip › Supplementary Figures/Supplementary Figure S10.pdf]

Supplementary Figure S10

Donor accessibility at five candidate-associated regions

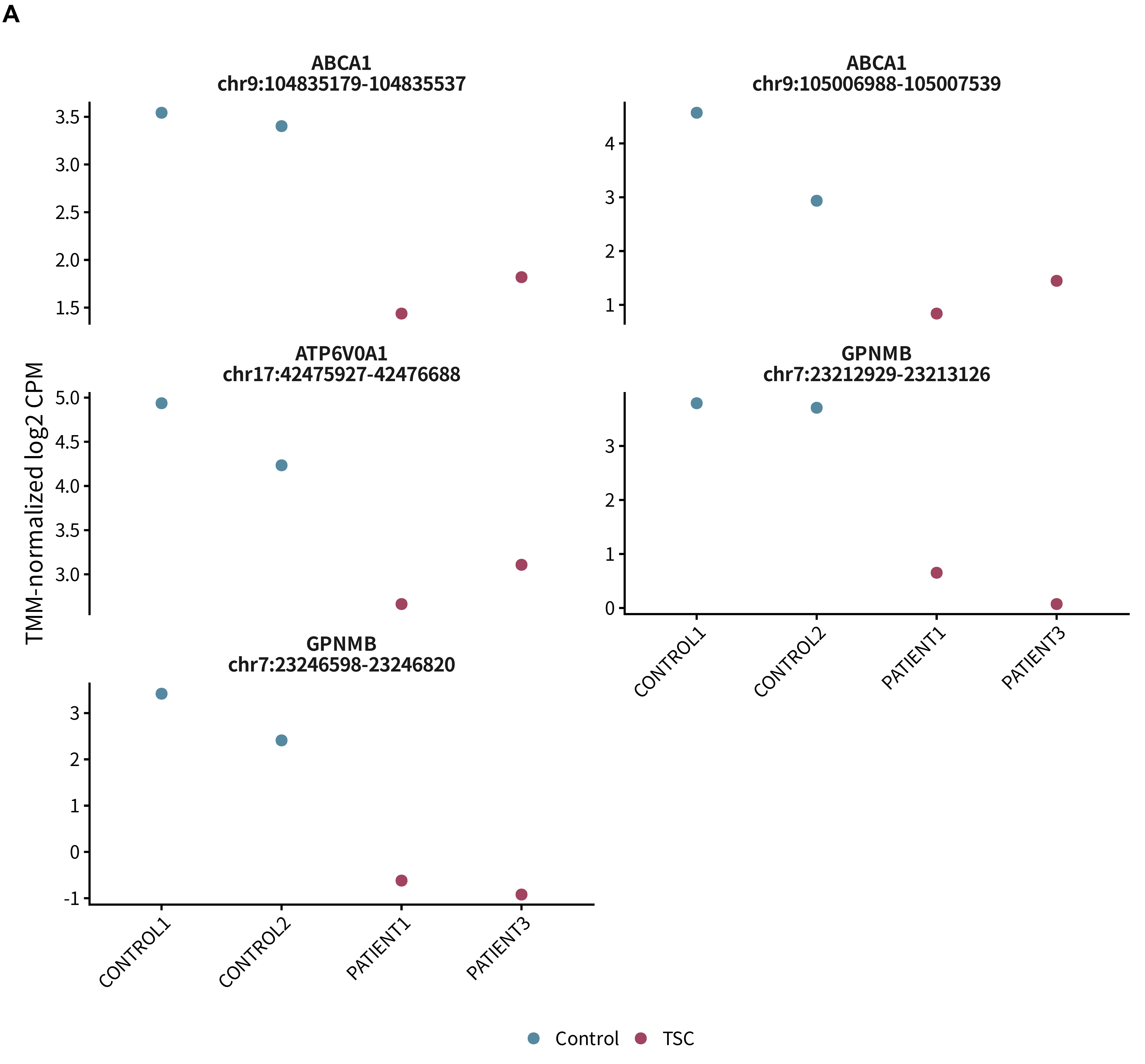

Figure legend

(A) TMM-normalized log2 counts per million at five decreased-accessibility DARs predicted by GREAT to be associated with ABCA1 (two regions), ATP6V0A1 (one), and GPNMB (two). Each point represents one donor pseudobulk (two Control and two TSC). Coordinates identify tested regions. None lies within the annotated TSS +/- 2 kb interval; links are predicted distal associations, not validated enhancers or demonstrated promoter closure.
