# Supplementary material for: Exploratory Single-Nucleus Transcriptomic and Chromatin Accessibility Profiling Indicates Microglial Remodeling in Tuberous Sclerosis Complex-Associated Epilepsy
Source: Biology (Basel). 2026 Nov 2;15(21):1976. doi: 10.3390/biology15211976 (PMC13762191; doi:10.3390/biology15211976)
Supplement: Supplementary file 1 [file biology-15-01976-s001.zip › Supplementary Figures/Supplementary Figure S11.pdf]

Supplementary Figure S11

Additional motif sequence logos and footprint profiles

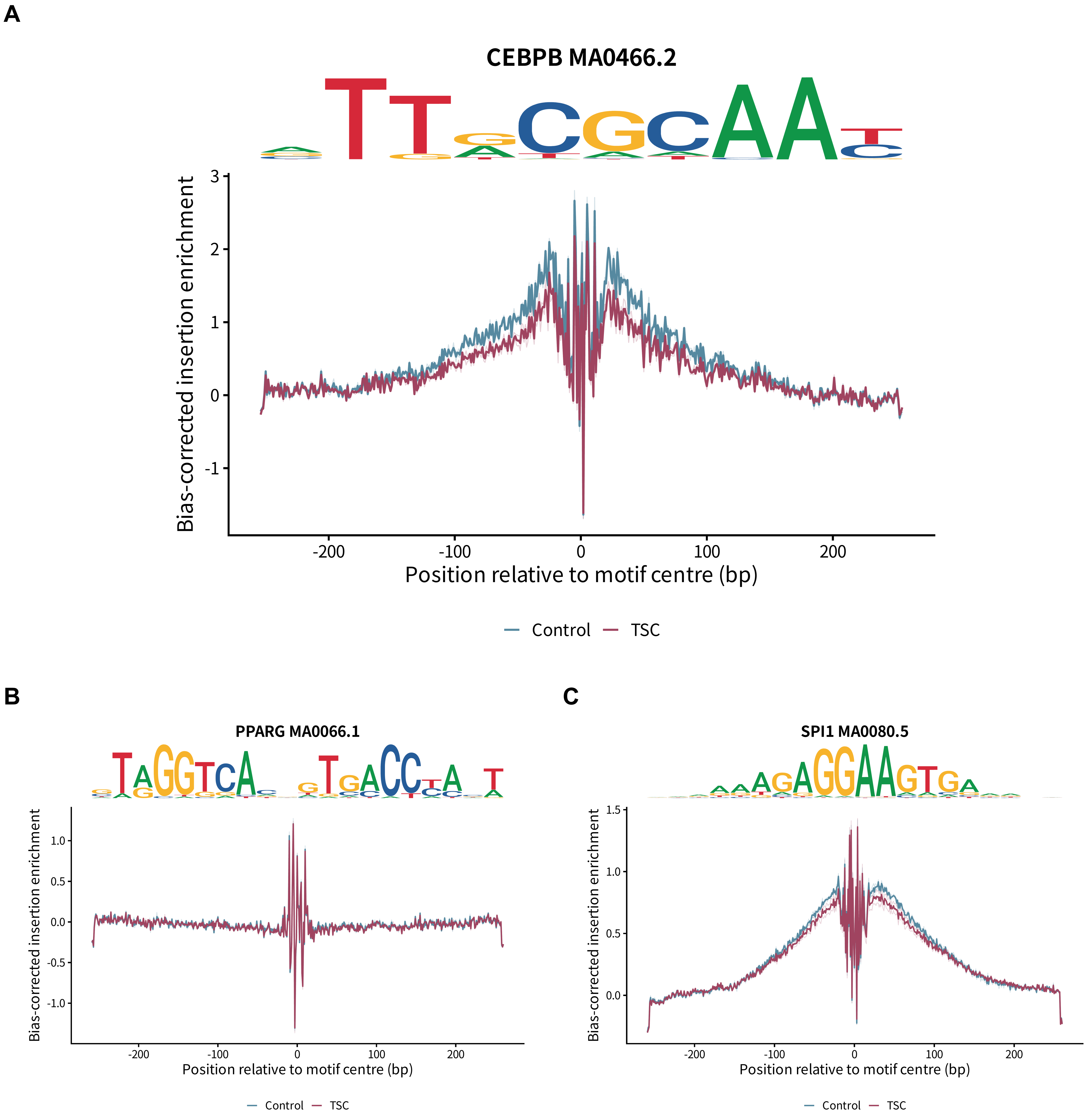

Figure legend

(A-C) CEBPB (A), PPARG (B), and SPI1 (C) sequence logos and footprint profiles. Logos show corresponding JASPAR motif preferences. Curves summarize normalized Tn5-bias-corrected insertion profiles around motif centers in the primary microglial ATAC population. Group curves are equally weighted donor means (two per group). Profiles are descriptive and do not establish direct factor-specific binding or functional activation.
