# Supplementary material for: Exploratory Single-Nucleus Transcriptomic and Chromatin Accessibility Profiling Indicates Microglial Remodeling in Tuberous Sclerosis Complex-Associated Epilepsy
Source: Biology (Basel). 2026 Nov 2;15(21):1976. doi: 10.3390/biology15211976 (PMC13762191; doi:10.3390/biology15211976)
Supplement: Supplementary file 1 [file biology-15-01976-s001.zip › Supplementary Figures/Supplementary Figure S12.pdf]

Supplementary Figure S12

TFEB and TFE3 footprint donor omission sensitivity

A

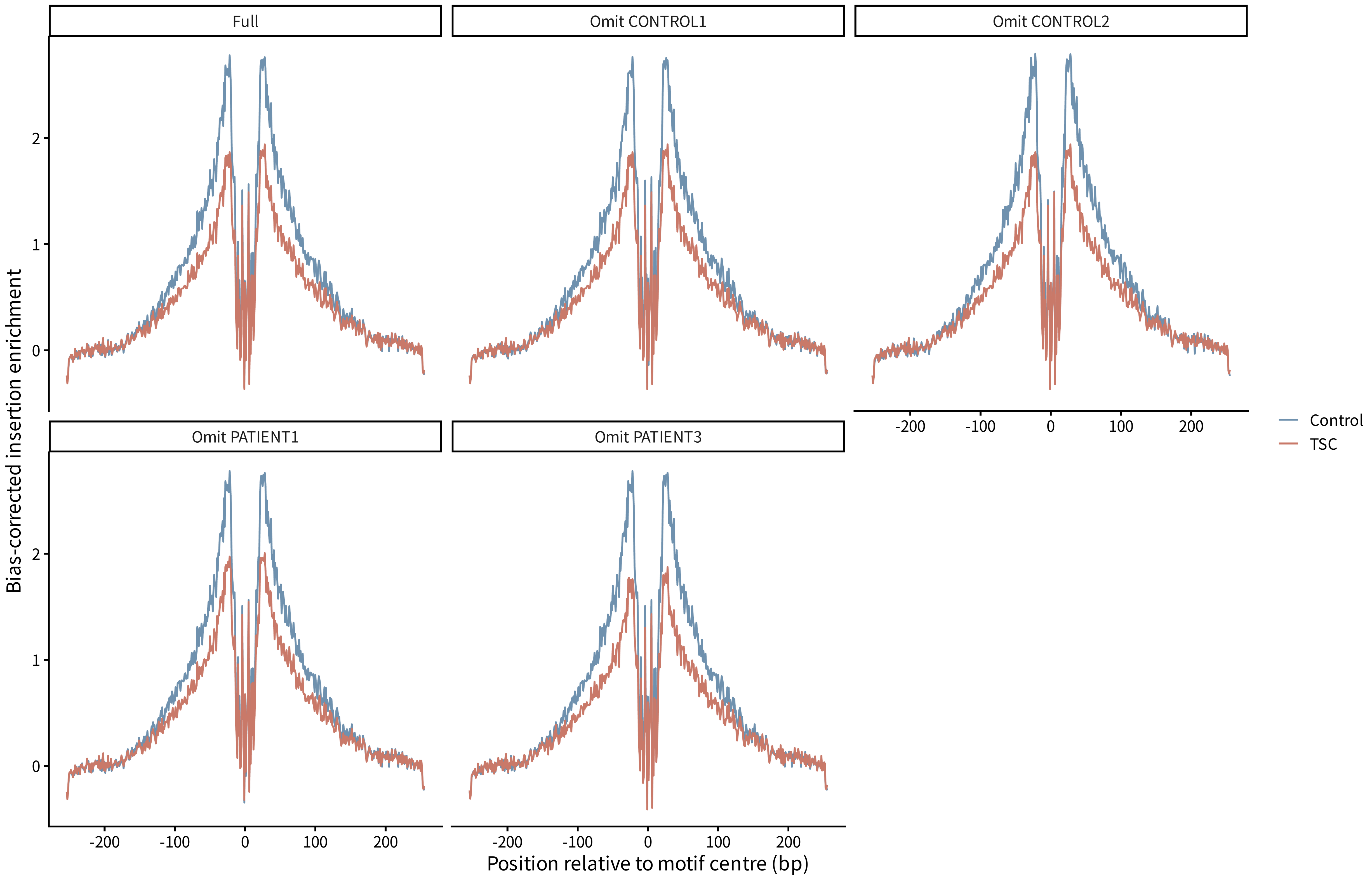

B

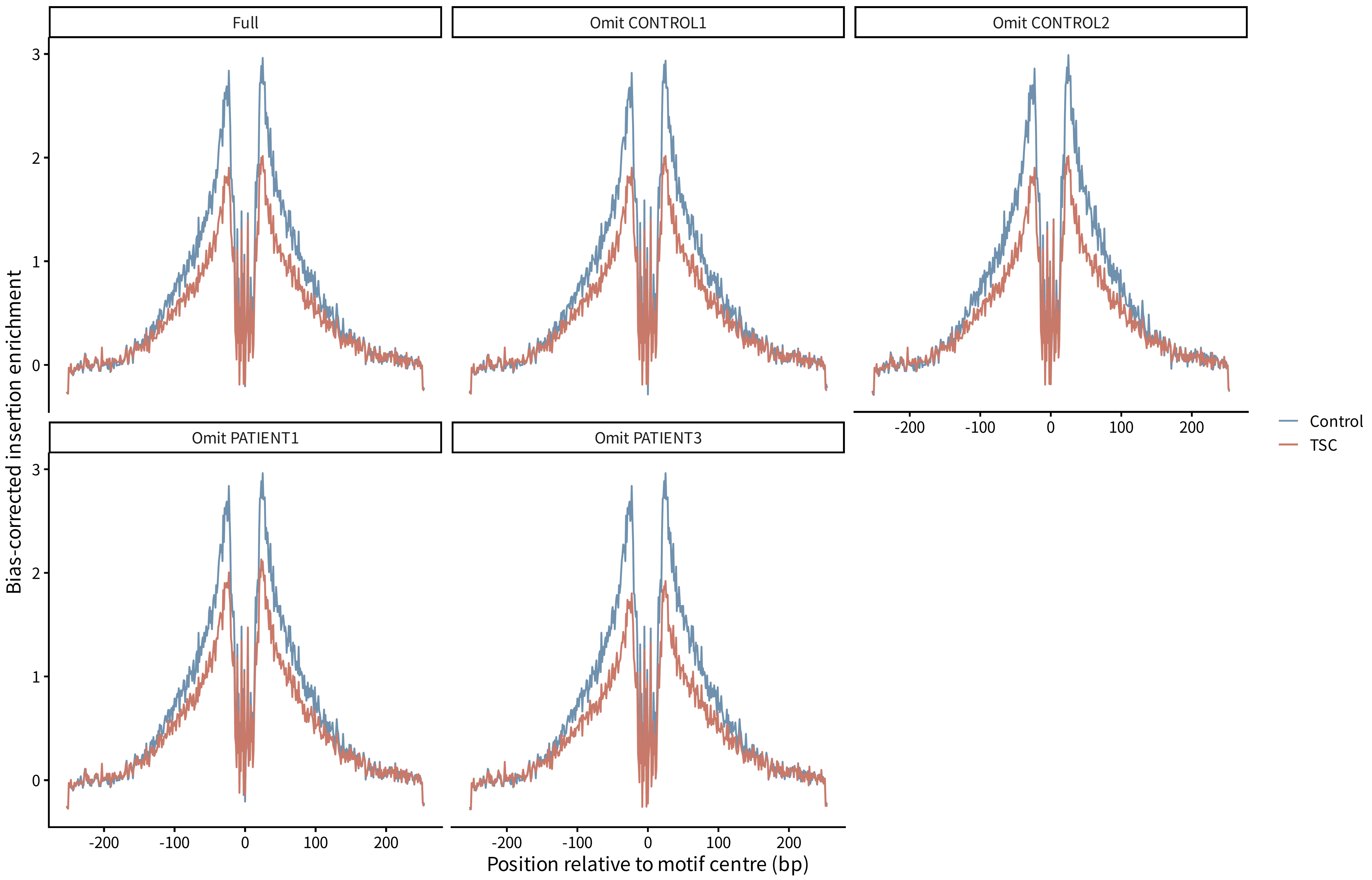

Figure legend

(A) TFEB MA0692.1 and (B) TFE3 MA0831.2 full and donor-omission bias-corrected insertion profiles at shared motif sites. Full group means use two donors per group; omission leaves one donor in the affected group. Donor omissions describe within-cohort sensitivity, not independent replication. Curves represent accessibility-related footprint patterns rather than direct evidence of factor-specific occupancy.
