# Supplementary material for: Exploratory Single-Nucleus Transcriptomic and Chromatin Accessibility Profiling Indicates Microglial Remodeling in Tuberous Sclerosis Complex-Associated Epilepsy
Source: Biology (Basel). 2026 Nov 2;15(21):1976. doi: 10.3390/biology15211976 (PMC13762191; doi:10.3390/biology15211976)
Supplement: Supplementary file 1 [file biology-15-01976-s001.zip › Supplementary Figures/Supplementary Figure S13.pdf]

Supplementary Figure S13

TFEB and TFE3 chromVAR donor omission sensitivity

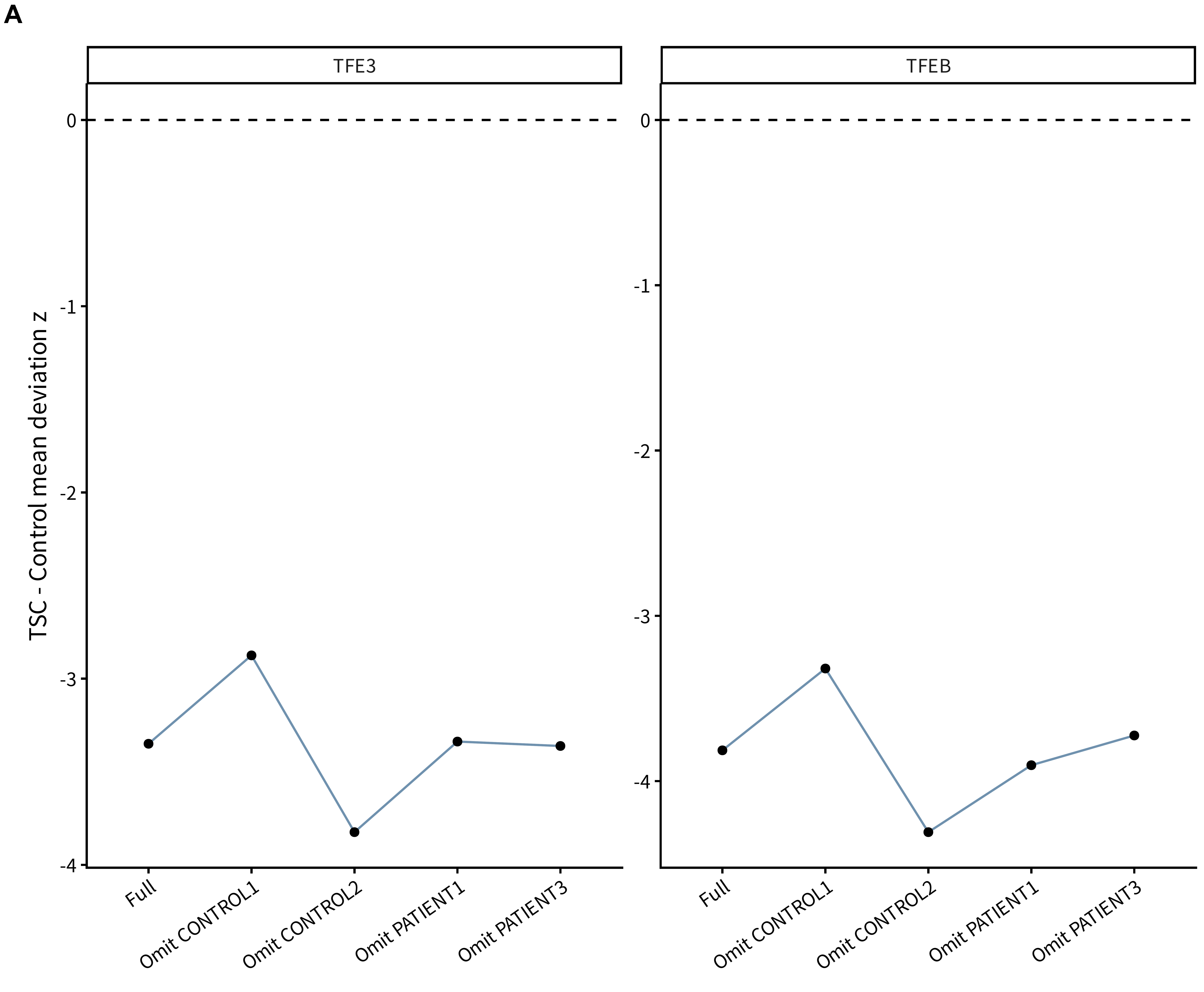

Figure legend

(A) Descriptive leave-one-donor-out changes in TFEB- and TFE3-associated donor-averaged chromVAR deviation z-scores. Full estimates use two donors per group; each omission leaves one donor in one group and two in the other. Retained effect direction is a sensitivity observation, not a statistically established change in transcription factor activity.
