# Supplementary material for: Exploratory Single-Nucleus Transcriptomic and Chromatin Accessibility Profiling Indicates Microglial Remodeling in Tuberous Sclerosis Complex-Associated Epilepsy
Source: Biology (Basel). 2026 Nov 2;15(21):1976. doi: 10.3390/biology15211976 (PMC13762191; doi:10.3390/biology15211976)
Supplement: Supplementary file 1 [file biology-15-01976-s001.zip › Supplementary Figures/Supplementary Figure S14.pdf]

Supplementary Figure S14

Candidate expression across individual surgical controls

A

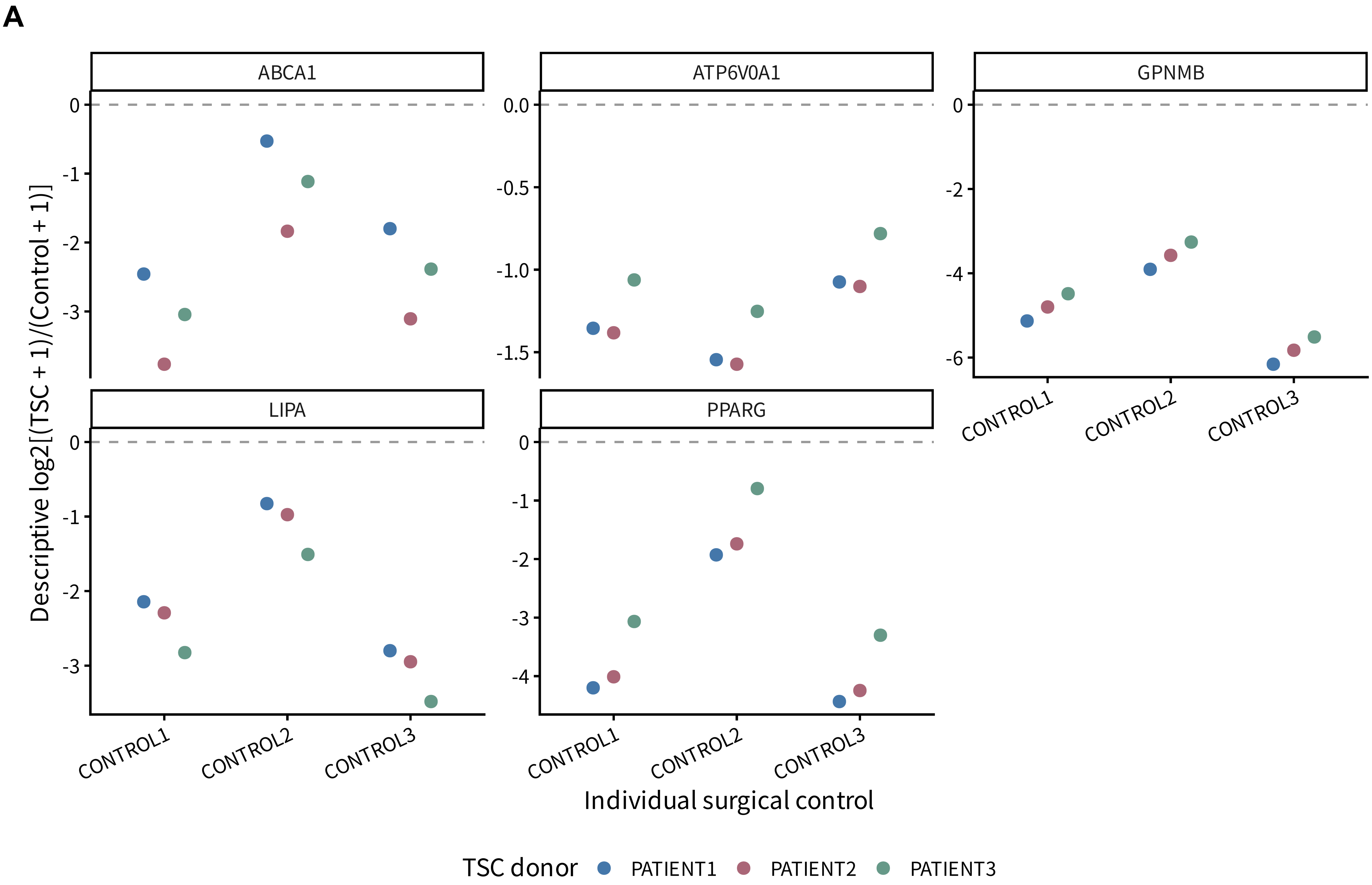

Figure legend

(A) Exploratory candidate-gene expression contrasts across individual surgical controls from the same six-person RNA cohort. Points represent log2[(normalized TSC expression + 1)/(normalized individual-Control expression + 1)], with colors identifying TSC donors. These descriptive ratios are not DESeq2 model coefficients. Consistency across individual controls does not provide independent replication or exclude shared effects of control pathology, medication, or tissue sampling.
