# Supplementary material for: Exploratory Single-Nucleus Transcriptomic and Chromatin Accessibility Profiling Indicates Microglial Remodeling in Tuberous Sclerosis Complex-Associated Epilepsy
Source: Biology (Basel). 2026 Nov 2;15(21):1976. doi: 10.3390/biology15211976 (PMC13762191; doi:10.3390/biology15211976)
Supplement: Supplementary file 1 [file biology-15-01976-s001.zip › Supplementary Figures/Supplementary Figure S15.pdf]

Supplementary Figure S15

RNA sequencing depth and differential-expression sensitivity

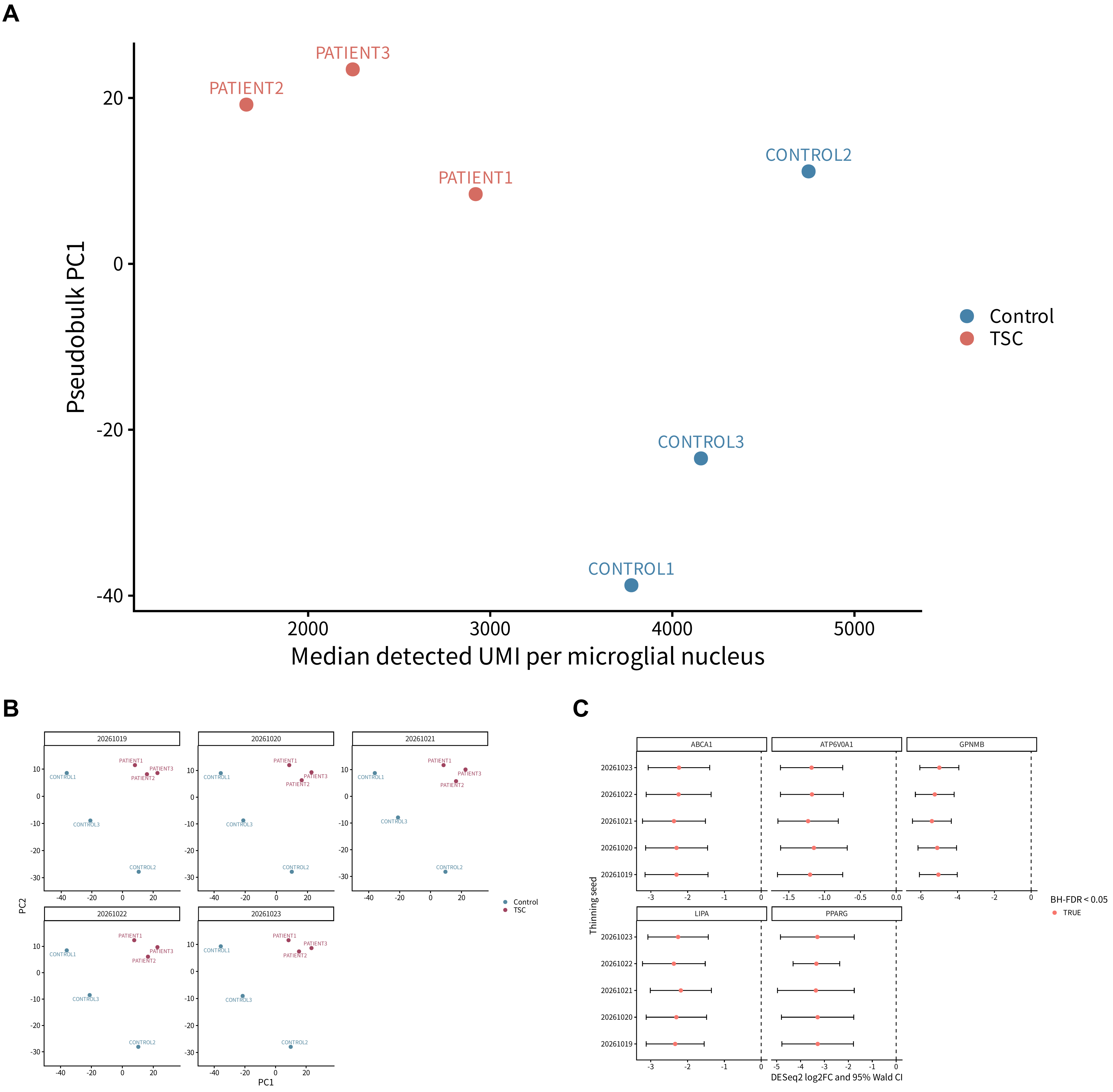

Figure legend

(A) Association between donor PC1 and median detected UMI depth. (B) Pseudobulk PCA across uniform-depth UMI-thinning runs. (C) Candidate-gene effect estimates across these runs. Donors remain the biological replicates (three per group). Repeated seeds evaluate subsampling sensitivity, not independent biological replication or removal of clinical confounding.
