# Supplementary material for: Exploratory Single-Nucleus Transcriptomic and Chromatin Accessibility Profiling Indicates Microglial Remodeling in Tuberous Sclerosis Complex-Associated Epilepsy
Source: Biology (Basel). 2026 Nov 2;15(21):1976. doi: 10.3390/biology15211976 (PMC13762191; doi:10.3390/biology15211976)
Supplement: Supplementary file 1 [file biology-15-01976-s001.zip › Supplementary Figures/Supplementary Figure S2.pdf]

Supplementary Figure S2

RNA donor quality control and pseudobulk ordination

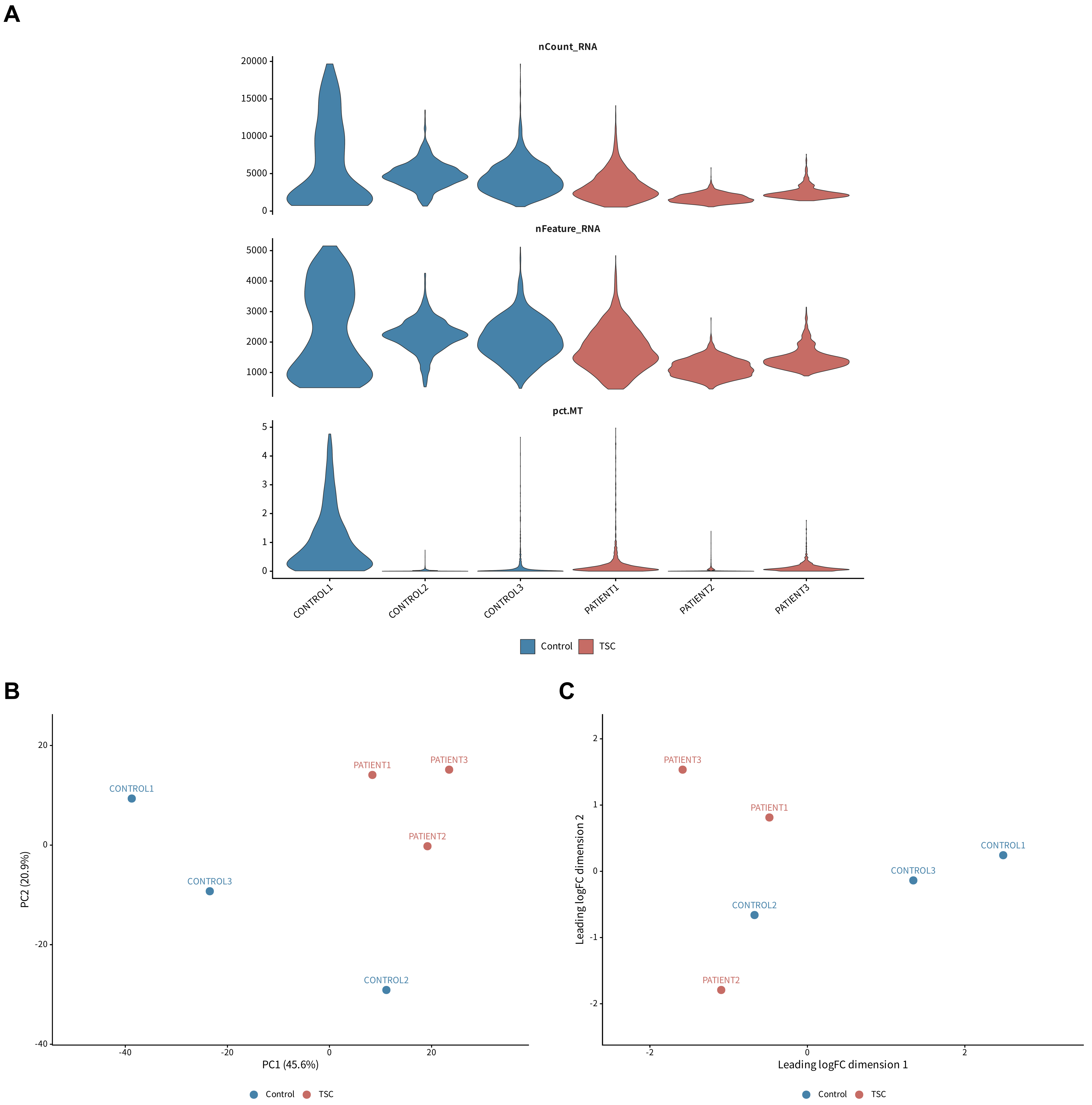

Figure legend

(A) Nucleus-level QC distributions in the strict 6,520-nucleus microglial population. (B) PCA of the six donor pseudobulks, using blind DESeq2 variance-stabilized counts and the 500 most variable genes, centered without scaling. (C) MDS using edgeR TMM-normalized leading log fold changes for 500 genes. Each point in B and C represents one independent donor (three Control and three TSC).
