# Supplementary material for: Exploratory Single-Nucleus Transcriptomic and Chromatin Accessibility Profiling Indicates Microglial Remodeling in Tuberous Sclerosis Complex-Associated Epilepsy
Source: Biology (Basel). 2026 Nov 2;15(21):1976. doi: 10.3390/biology15211976 (PMC13762191; doi:10.3390/biology15211976)
Supplement: Supplementary file 1 [file biology-15-01976-s001.zip › Supplementary Figures/Supplementary Figure S3.pdf]

Supplementary Figure S3

Candidate RNA expression and donor omission sensitivity

A

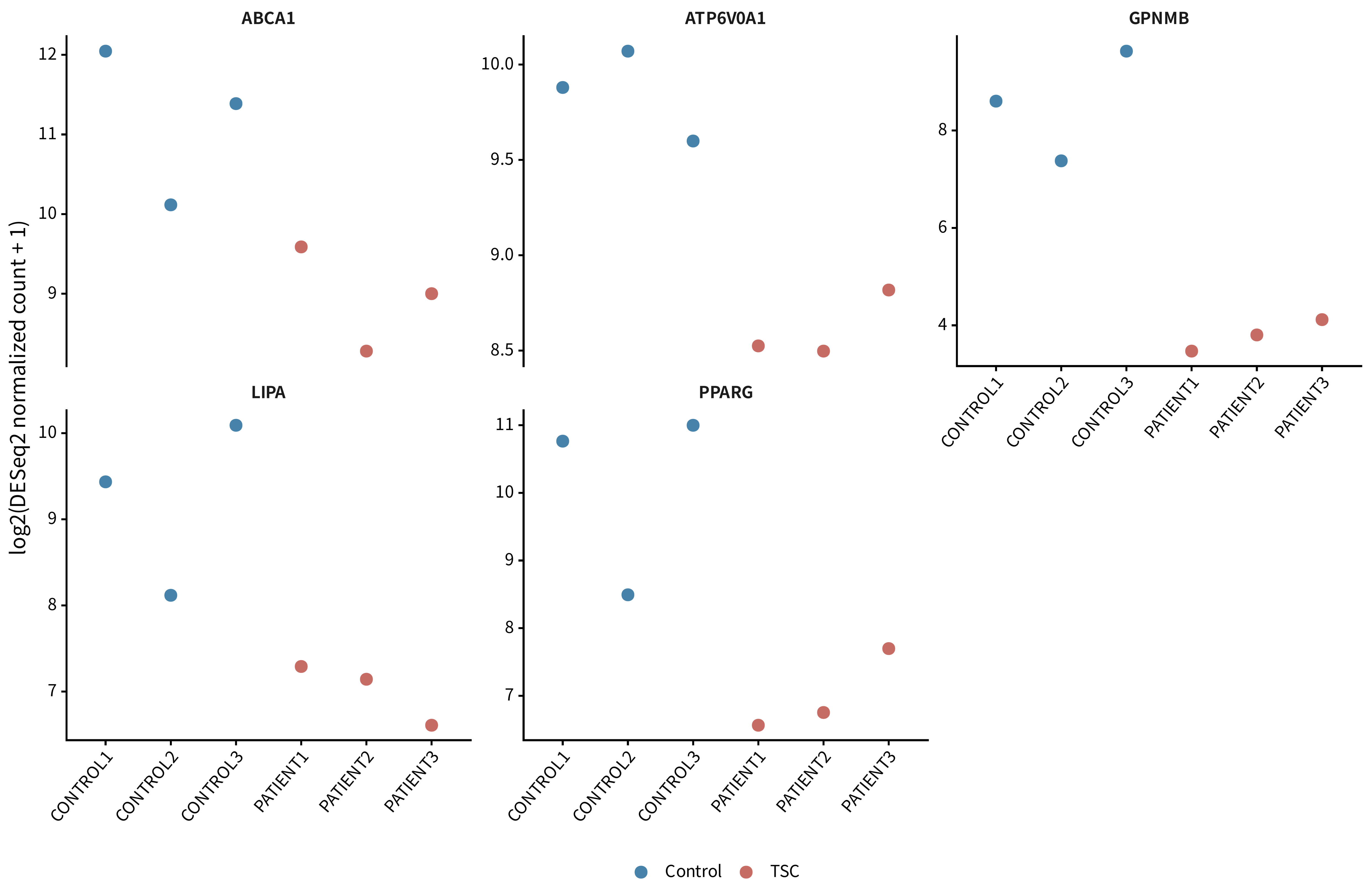

B

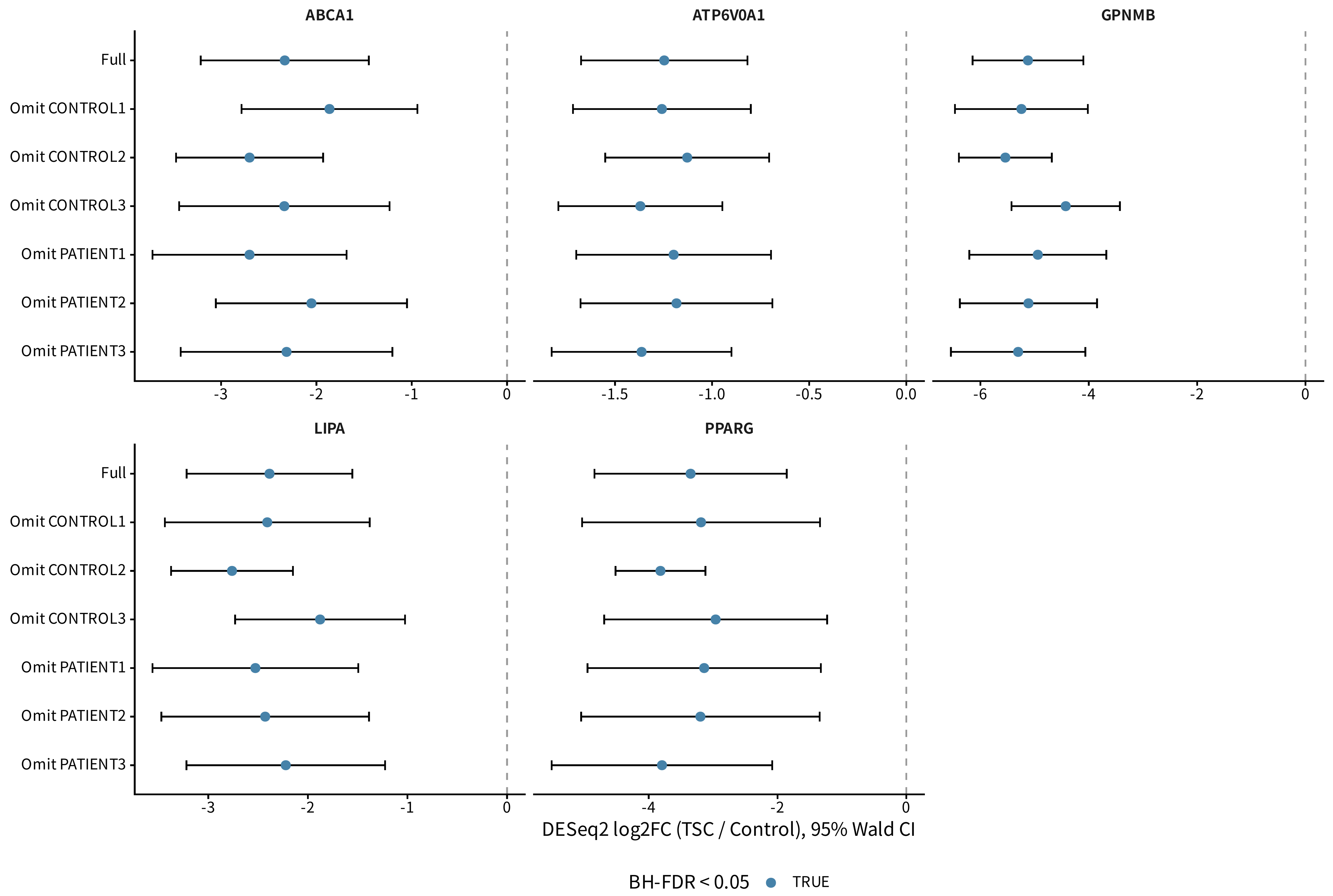

Figure legend

(A) Donor-level LIPA, ABCA1, ATP6V0A1, GPNMB, and PPARG expression as log2(DESeq2 size-factor-normalized pseudobulk counts + 1). (B) Full-model and leave-one-donor-out DESeq2 log2 fold changes with approximate intervals of estimate +/- 1.96 standard errors. Positive values denote higher expression in TSC. Analyses use the strict microglial population and a fixed gene universe. Full models include three donors per group; each omission removes one donor. These are sensitivity analyses of the same cohort, not independent replication.
