# Supplementary material for: Exploratory Single-Nucleus Transcriptomic and Chromatin Accessibility Profiling Indicates Microglial Remodeling in Tuberous Sclerosis Complex-Associated Epilepsy
Source: Biology (Basel). 2026 Nov 2;15(21):1976. doi: 10.3390/biology15211976 (PMC13762191; doi:10.3390/biology15211976)
Supplement: Supplementary file 1 [file biology-15-01976-s001.zip › Supplementary Figures/Supplementary Figure S4.pdf]

Supplementary Figure S4

Gene-set and pathway-level donor omission sensitivity

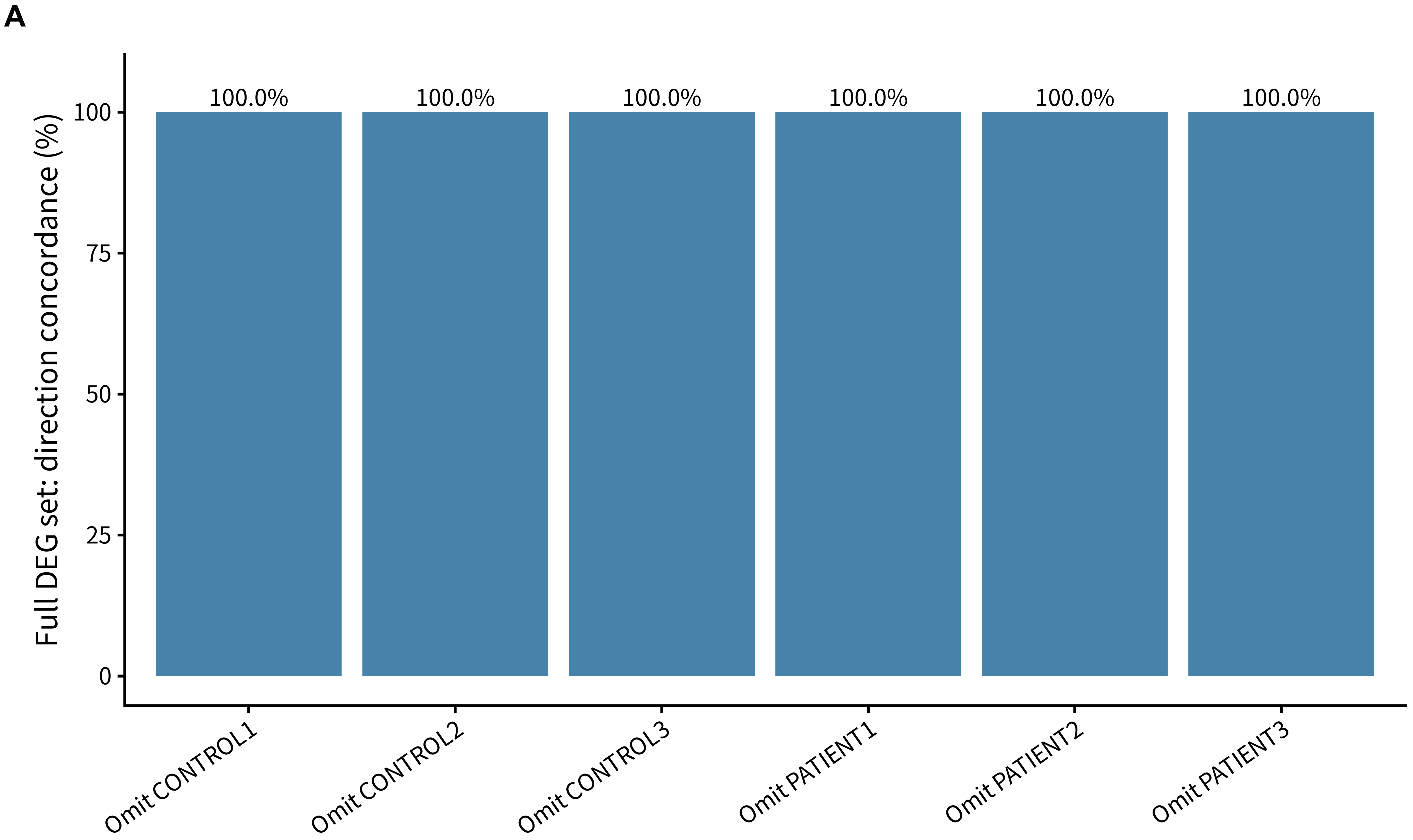

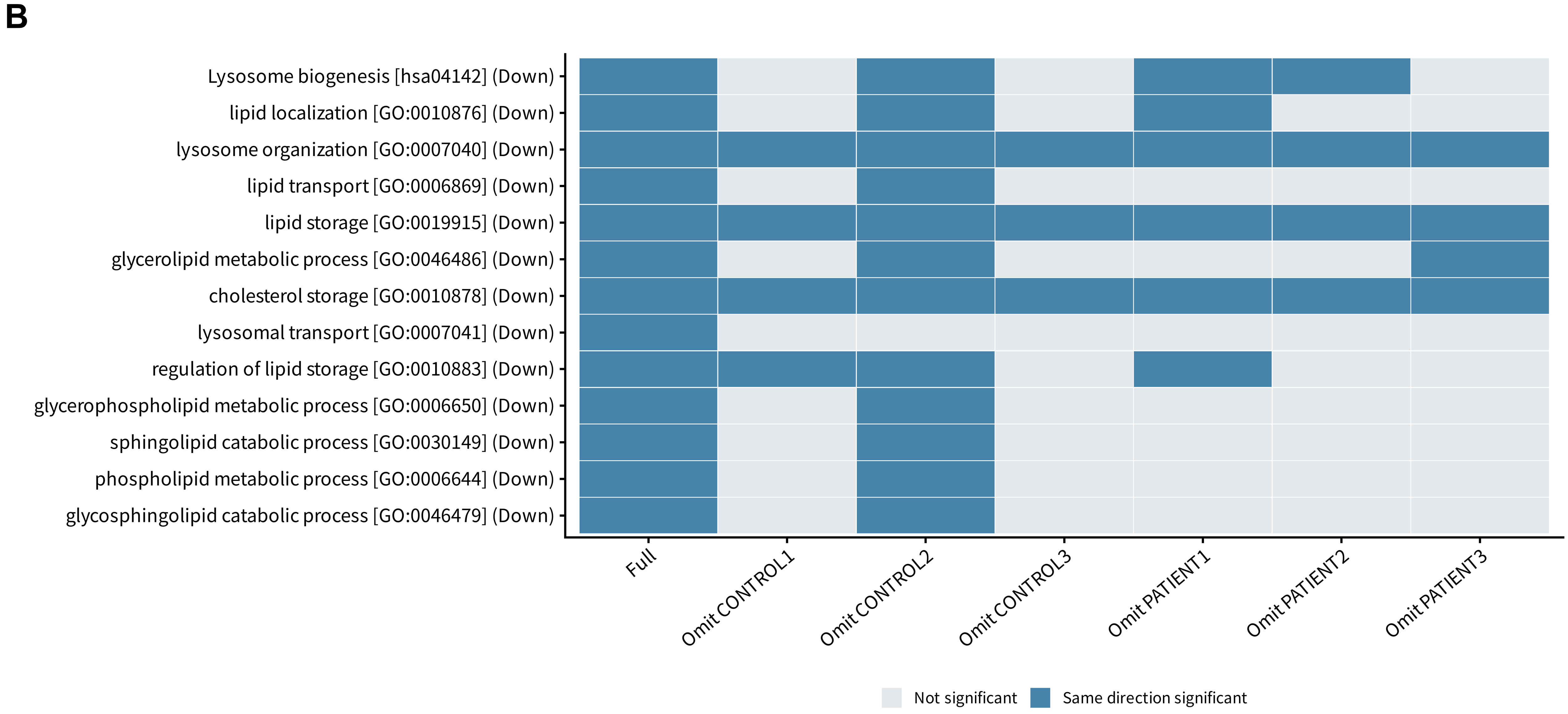

Figure legend

(A) Direction concordance of the full-model microglial DEG set across donor-omission fits. (B) Retention of selected lipid- and lysosome-related ORA results. RNA DEGs require DESeq2 BH-FDR < 0.05 and absolute log2FC >= 0.25; ORA significance uses BH-FDR < 0.05 within each directional database analysis. Directional enrichment denotes membership in upregulated or downregulated gene lists, not measured pathway activity. Loss of enrichment significance is distinct from reversal of gene-level effects.
