# Supplementary material for: Exploratory Single-Nucleus Transcriptomic and Chromatin Accessibility Profiling Indicates Microglial Remodeling in Tuberous Sclerosis Complex-Associated Epilepsy
Source: Biology (Basel). 2026 Nov 2;15(21):1976. doi: 10.3390/biology15211976 (PMC13762191; doi:10.3390/biology15211976)
Supplement: Supplementary file 1 [file biology-15-01976-s001.zip › Supplementary Figures/Supplementary Figure S5.pdf]

Supplementary Figure S5

Exploratory state coverage and sensitivity to UMI thinning

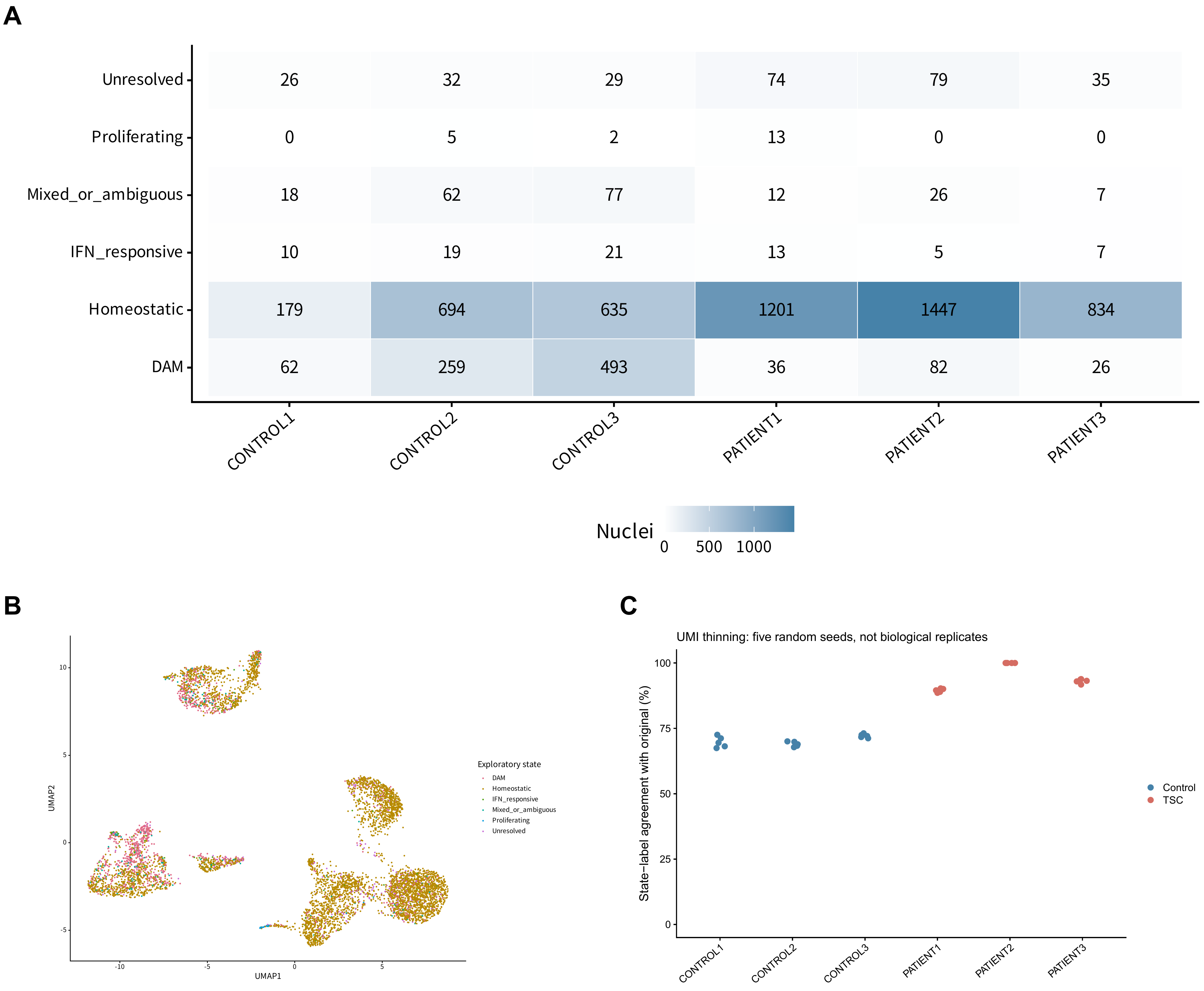

Figure legend

(A) Donor coverage of provisional microglial states. (B) Exploratory module-score state assignments on the pre-exclusion microglial embedding. (C) Agreement of assignments after UMI thinning. States do not establish discrete functional lineages. Missing donor-state combinations were not replaced by zero-expression pseudobulks. Within-state tests required at least 10 nuclei from every donor; state-balanced analysis was restricted to states with sufficient shared coverage. Repeated thinning seeds are technical sensitivity runs, not additional donors.
