# Supplementary material for: Exploratory Single-Nucleus Transcriptomic and Chromatin Accessibility Profiling Indicates Microglial Remodeling in Tuberous Sclerosis Complex-Associated Epilepsy
Source: Biology (Basel). 2026 Nov 2;15(21):1976. doi: 10.3390/biology15211976 (PMC13762191; doi:10.3390/biology15211976)
Supplement: Supplementary file 1 [file biology-15-01976-s001.zip › Supplementary Figures/Supplementary Figure S6.pdf]

Supplementary Figure S6

Preranked GSEA of strict microglial donor-level RNA differences

A

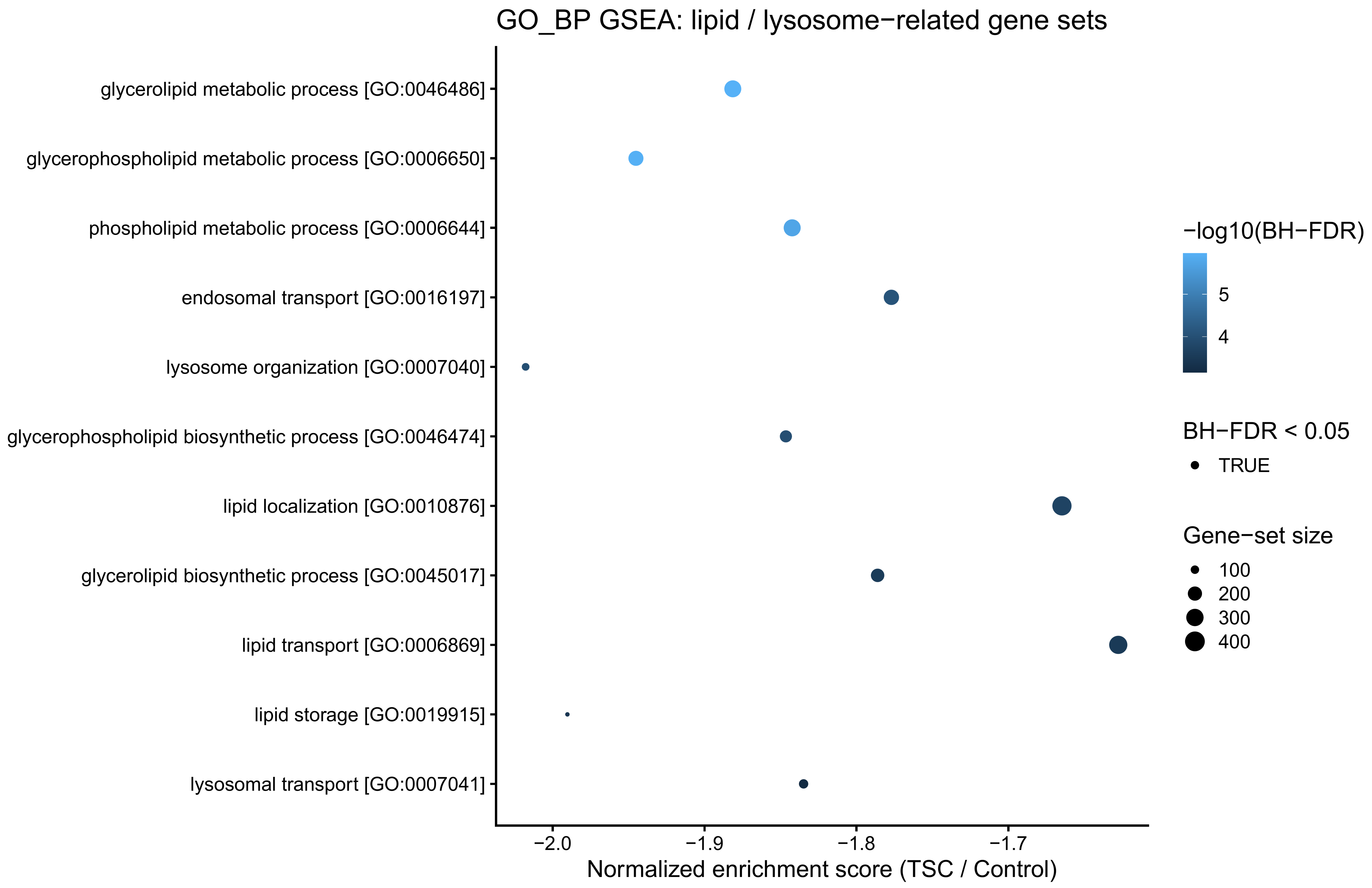

B

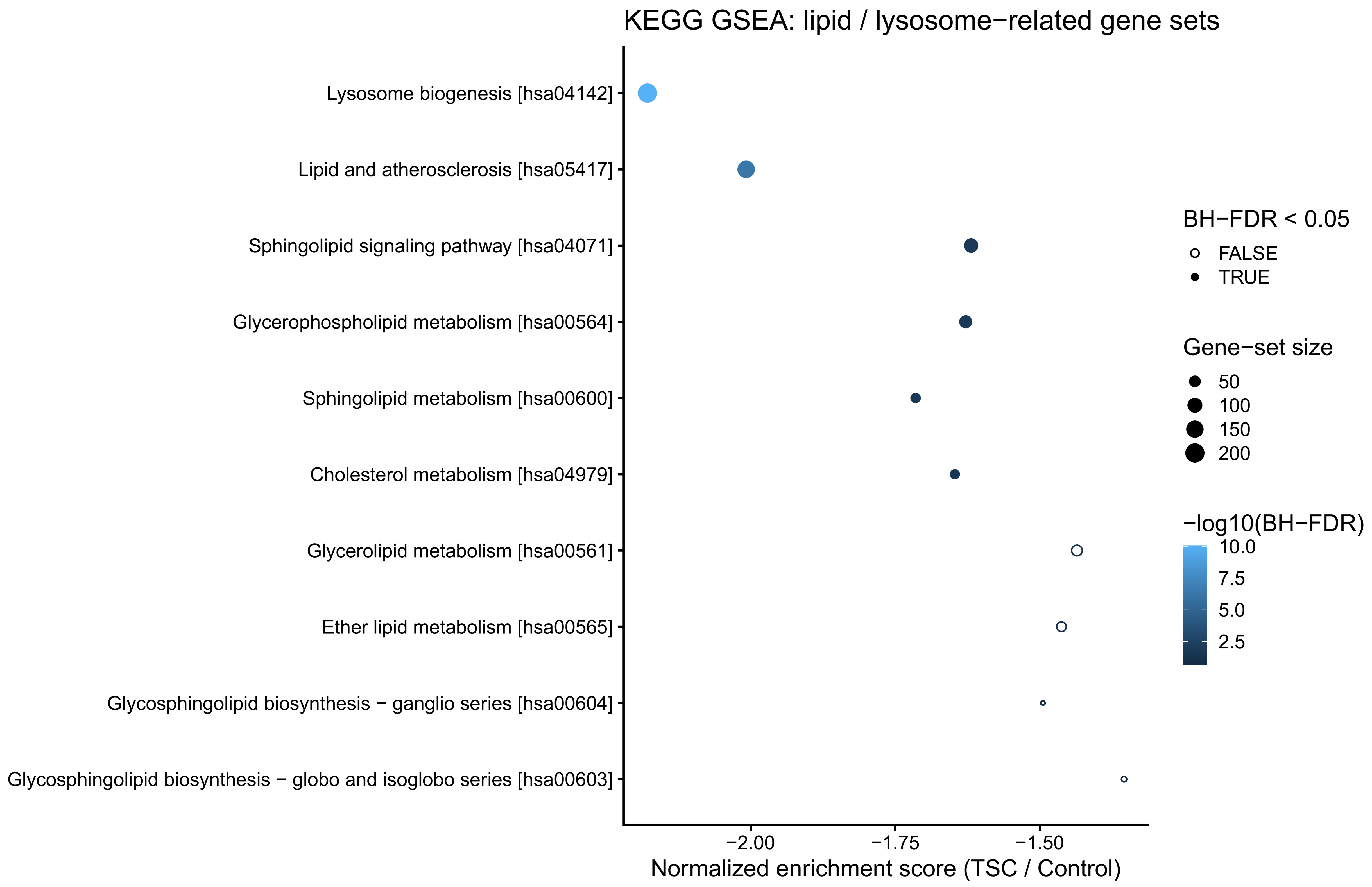

Supplementary Figure S6 (continued)

Preranked GSEA of strict microglial donor-level RNA differences

C

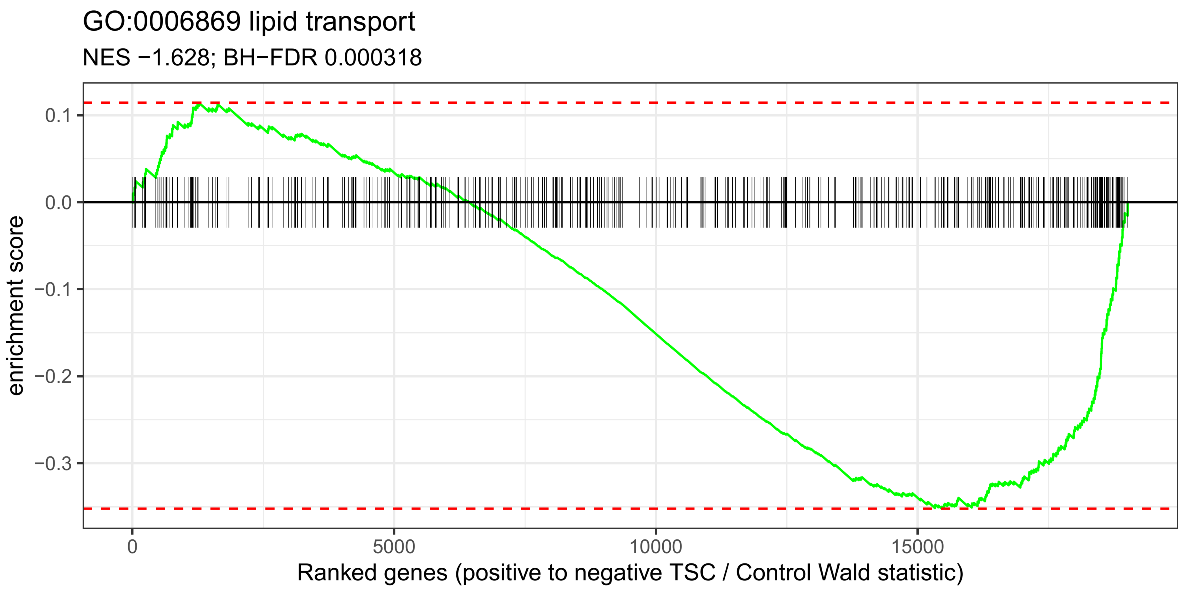

D

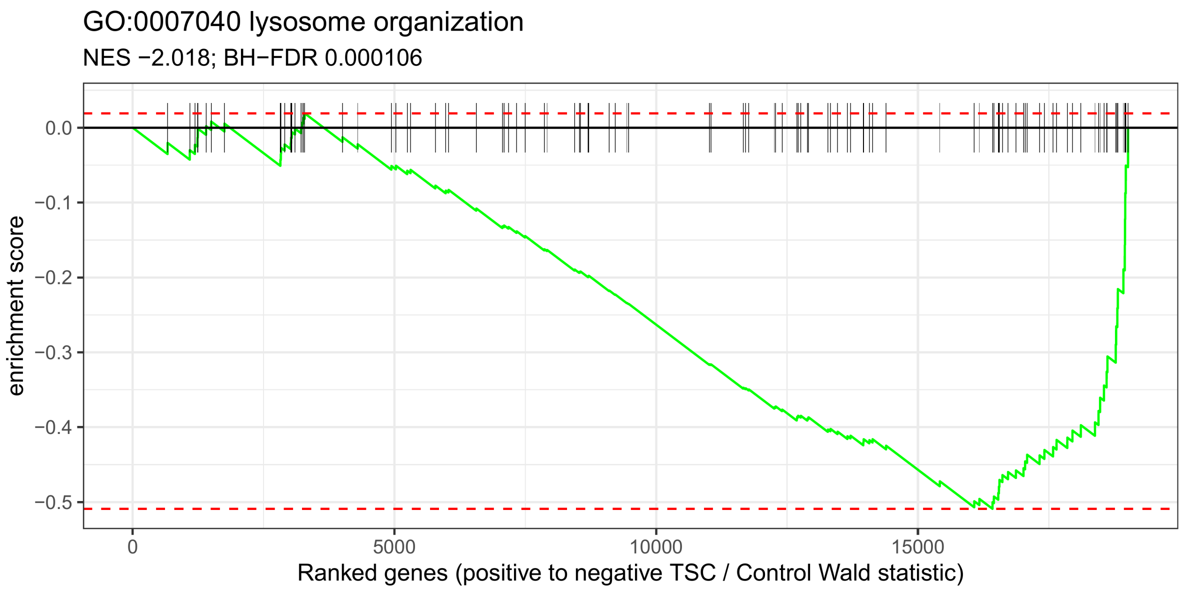

E

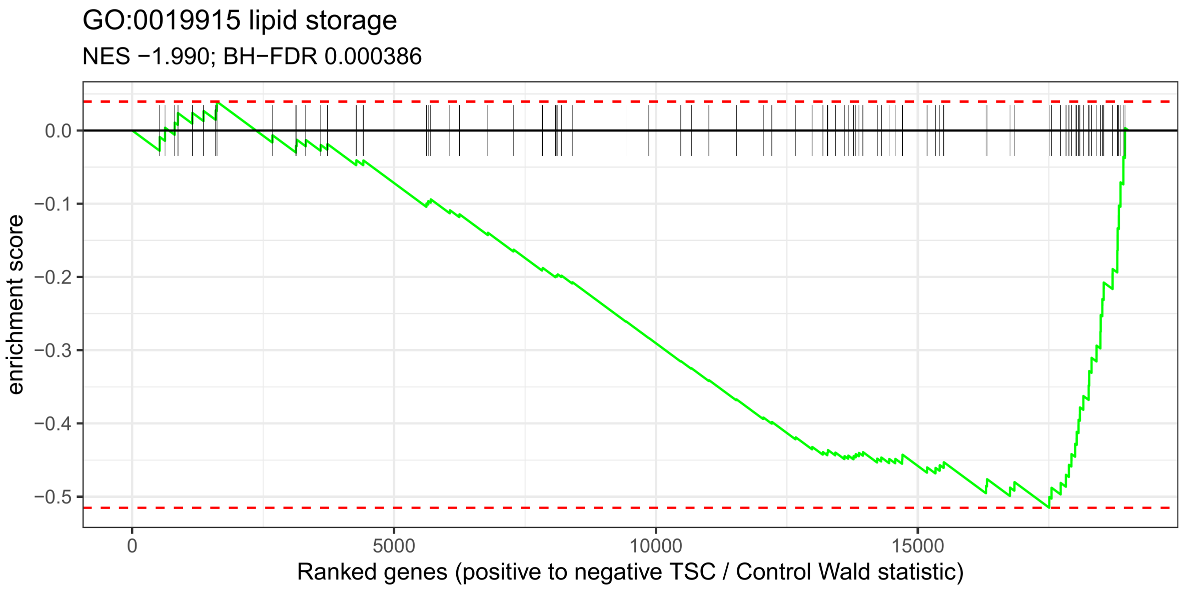

F

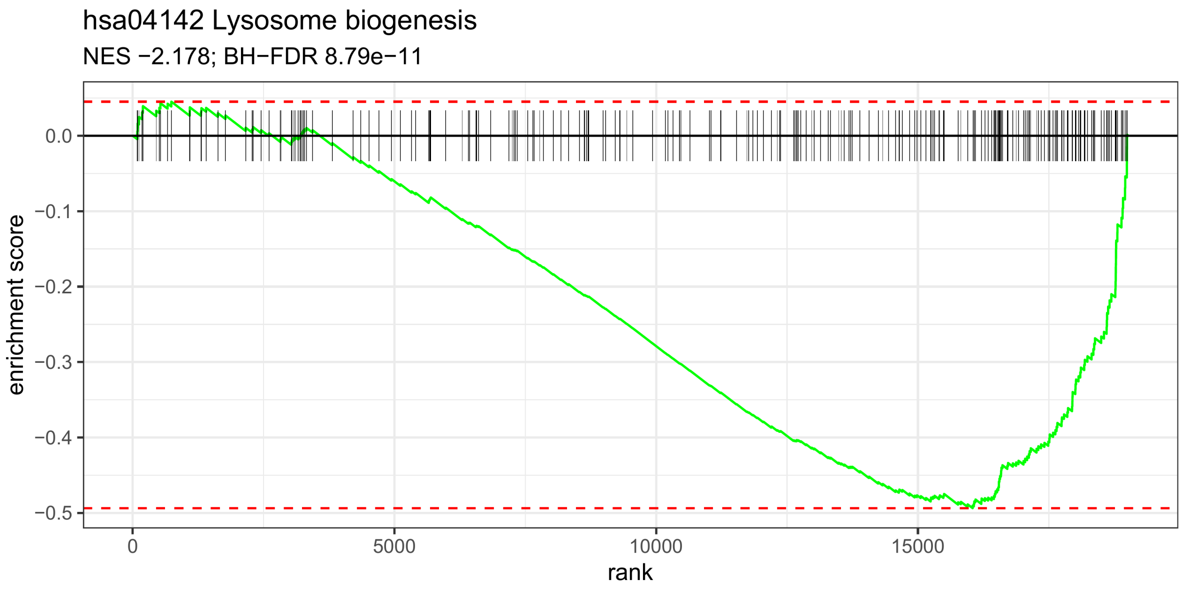

G

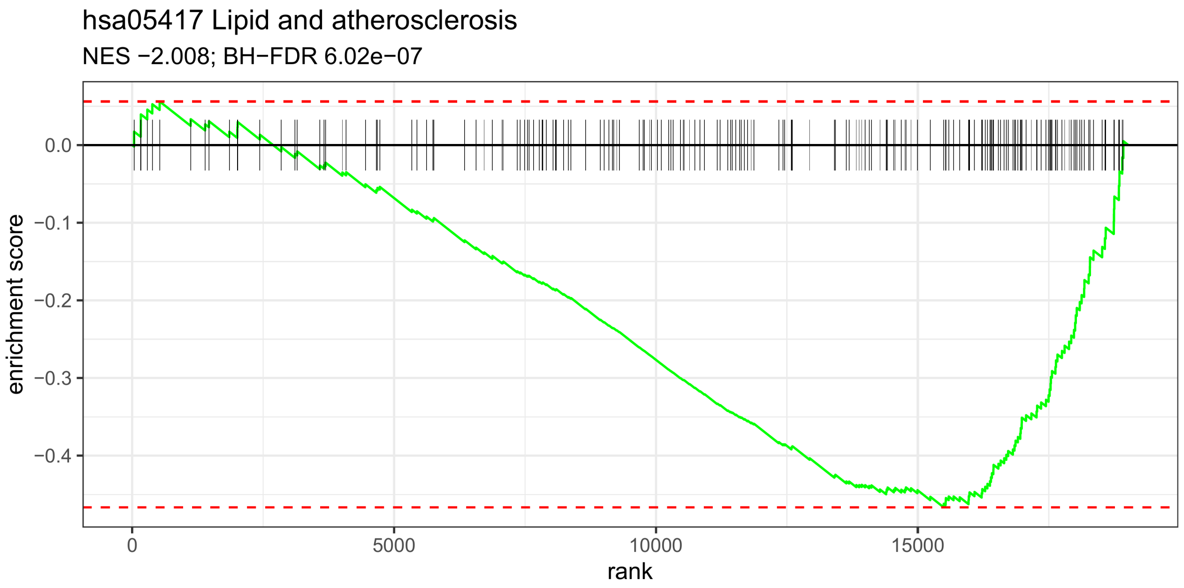

Figure legend

(A, B) Selected negatively enriched lipid- and lysosome-related GO Biological Process (A) and KEGG (B) terms. (C-G) Running enrichment scores for lipid transport (C), lysosome organization (D), lipid storage (E), KEGG hsa04142 (F), and lipid and atherosclerosis (G). Gene sets were tested using fgsea 1.32.4 on the complete finite DESeq2 Wald-statistic ranking after exclusion of 109 BAM-like candidates (three donors per group), with 15-500 ranked genes per set. Negative NES indicates enrichment toward lower expression in TSC. BH-FDR was calculated across all tested terms separately within GO BP and KEGG. Autophagy-related terms are omitted only from this focused display; the complete unchanged results are in Supplementary Table S7. This is a complementary analysis of the same cohort, not independent validation or direct measurement of pathway function.
