# Supplementary material for: Exploratory Single-Nucleus Transcriptomic and Chromatin Accessibility Profiling Indicates Microglial Remodeling in Tuberous Sclerosis Complex-Associated Epilepsy
Source: Biology (Basel). 2026 Nov 2;15(21):1976. doi: 10.3390/biology15211976 (PMC13762191; doi:10.3390/biology15211976)
Supplement: Supplementary file 1 [file biology-15-01976-s001.zip › Supplementary Figures/Supplementary Figure S7.pdf]

Supplementary Figure S7

Marker-based reassessment of microglial identity

A

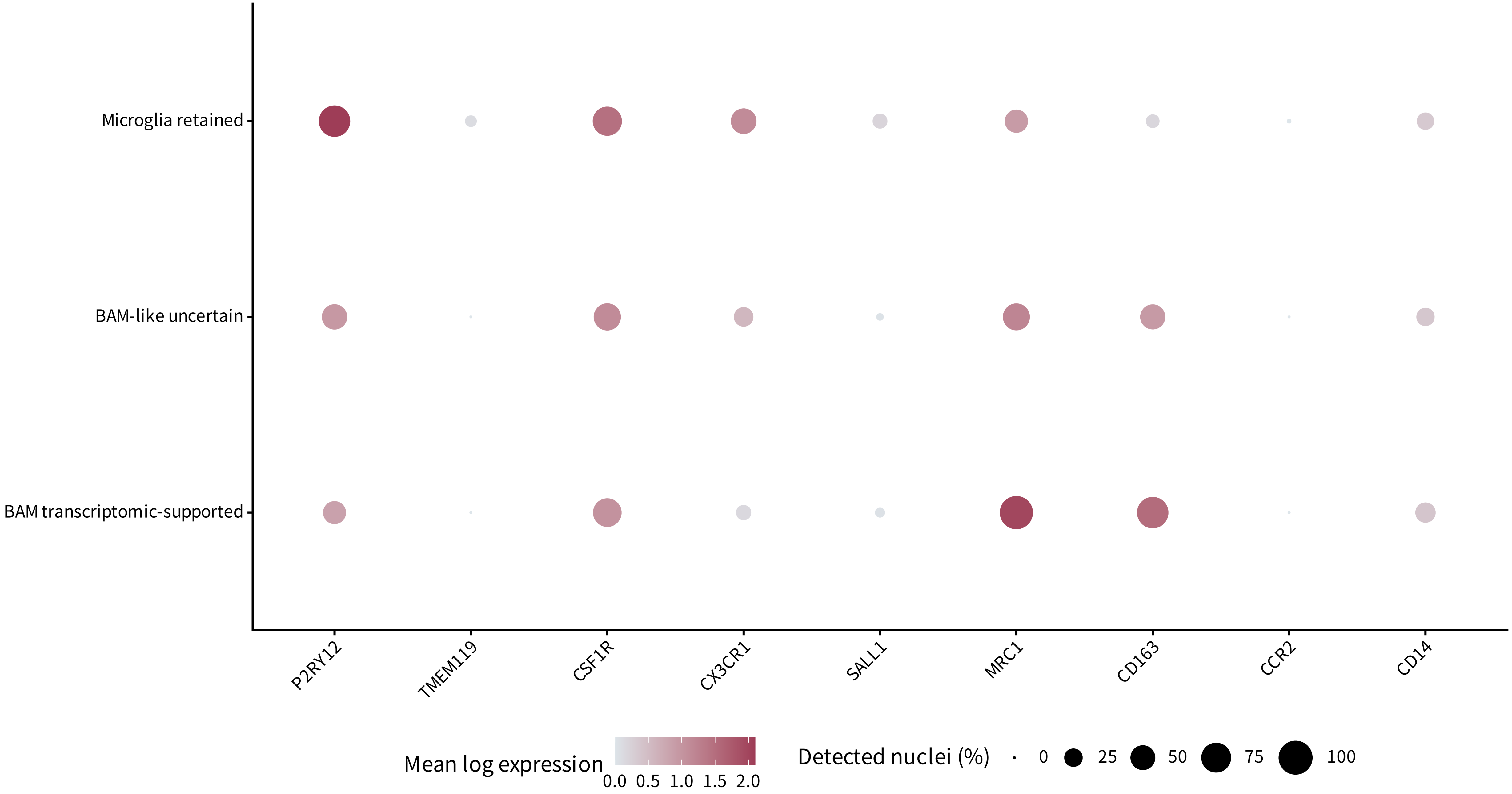

Figure legend

(A) Expression of P2RY12, TMEM119, CSF1R, CX3CR1, SALL1, MRC1, CD163, CCR2, and CD14 across operational microglial identity classes. Dot size denotes the percentage of nuclei with detected expression; color denotes mean log-normalized expression. The starting population contained 6,629 nuclei, including 19 supported and 90 uncertain BAM-like candidates. Exclusion of all 109 candidates retained 6,520 microglial nuclei. These are transcriptomic assignments, not lineage-validated identities; no supported monocyte candidates were identified.
