# Supplementary material for: Exploratory Single-Nucleus Transcriptomic and Chromatin Accessibility Profiling Indicates Microglial Remodeling in Tuberous Sclerosis Complex-Associated Epilepsy
Source: Biology (Basel). 2026 Nov 2;15(21):1976. doi: 10.3390/biology15211976 (PMC13762191; doi:10.3390/biology15211976)
Supplement: Supplementary file 1 [file biology-15-01976-s001.zip › Supplementary Figures/Supplementary Figure S8.pdf]

Supplementary Figure S8

GREAT and motif enrichment sensitivity to depth and QC

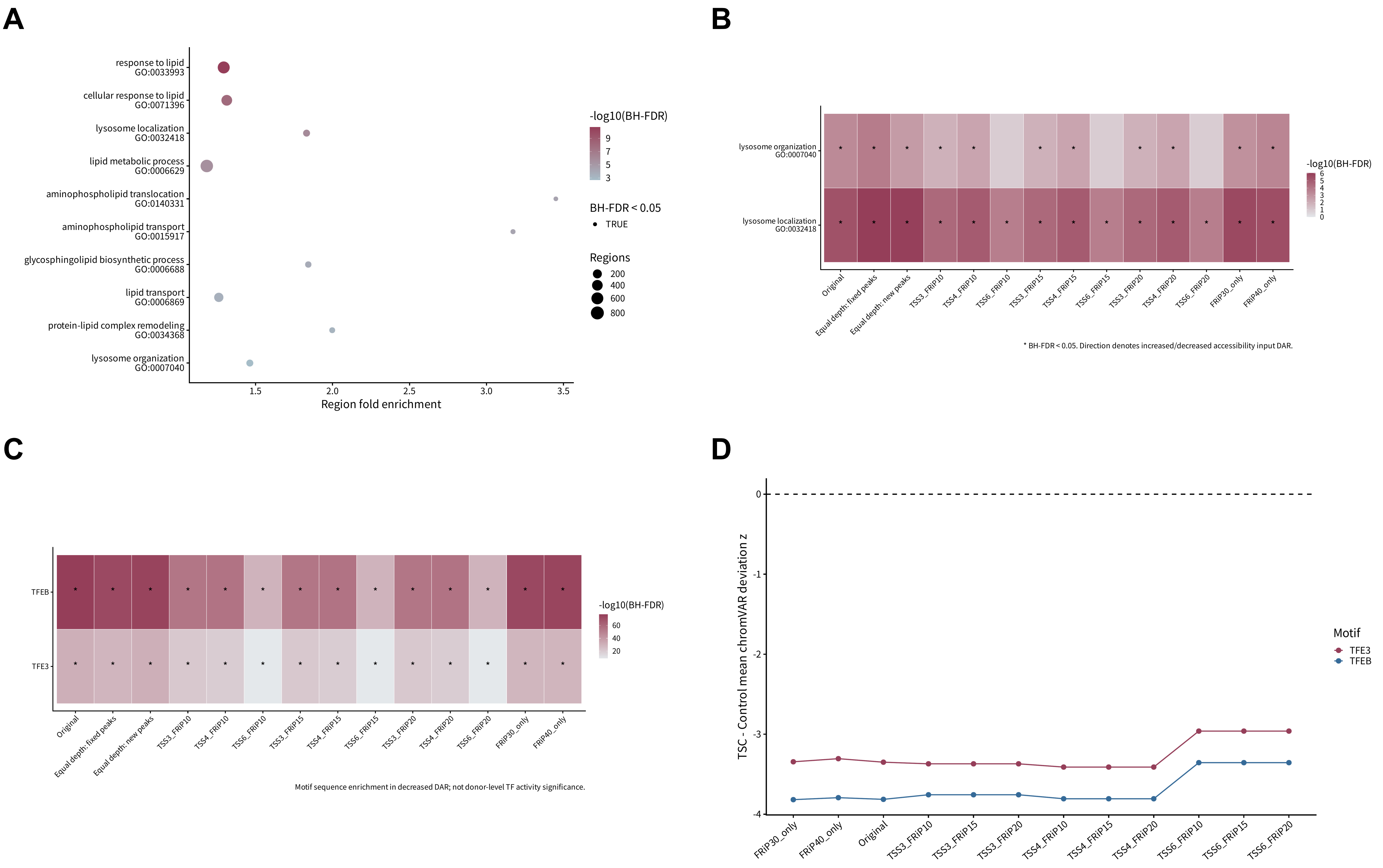

Figure legend

(A) Selected lipid- and lysosome-related GREAT results for the equal-depth rebuilt consensus. (B) Selected GREAT terms across depth/QC scenarios. (C) TFEB/TFE3 motif enrichment across scenarios. (D) chromVAR effect directions under the indicated QC settings. Scenario-specific nucleus counts and tested-region backgrounds are provided in Supplementary Table S8. Region-set motif enrichment is distinct from a donor-level test of transcription factor activity; not all terms remain significant under every setting.
