# Supplementary material for: Exploratory Single-Nucleus Transcriptomic and Chromatin Accessibility Profiling Indicates Microglial Remodeling in Tuberous Sclerosis Complex-Associated Epilepsy
Source: Biology (Basel). 2026 Nov 2;15(21):1976. doi: 10.3390/biology15211976 (PMC13762191; doi:10.3390/biology15211976)
Supplement: Supplementary file 1 [file biology-15-01976-s001.zip › Supplementary Figures/Supplementary Figure S9.pdf]

Supplementary Figure S9

Concordance of microglial ATAC differential-accessibility models

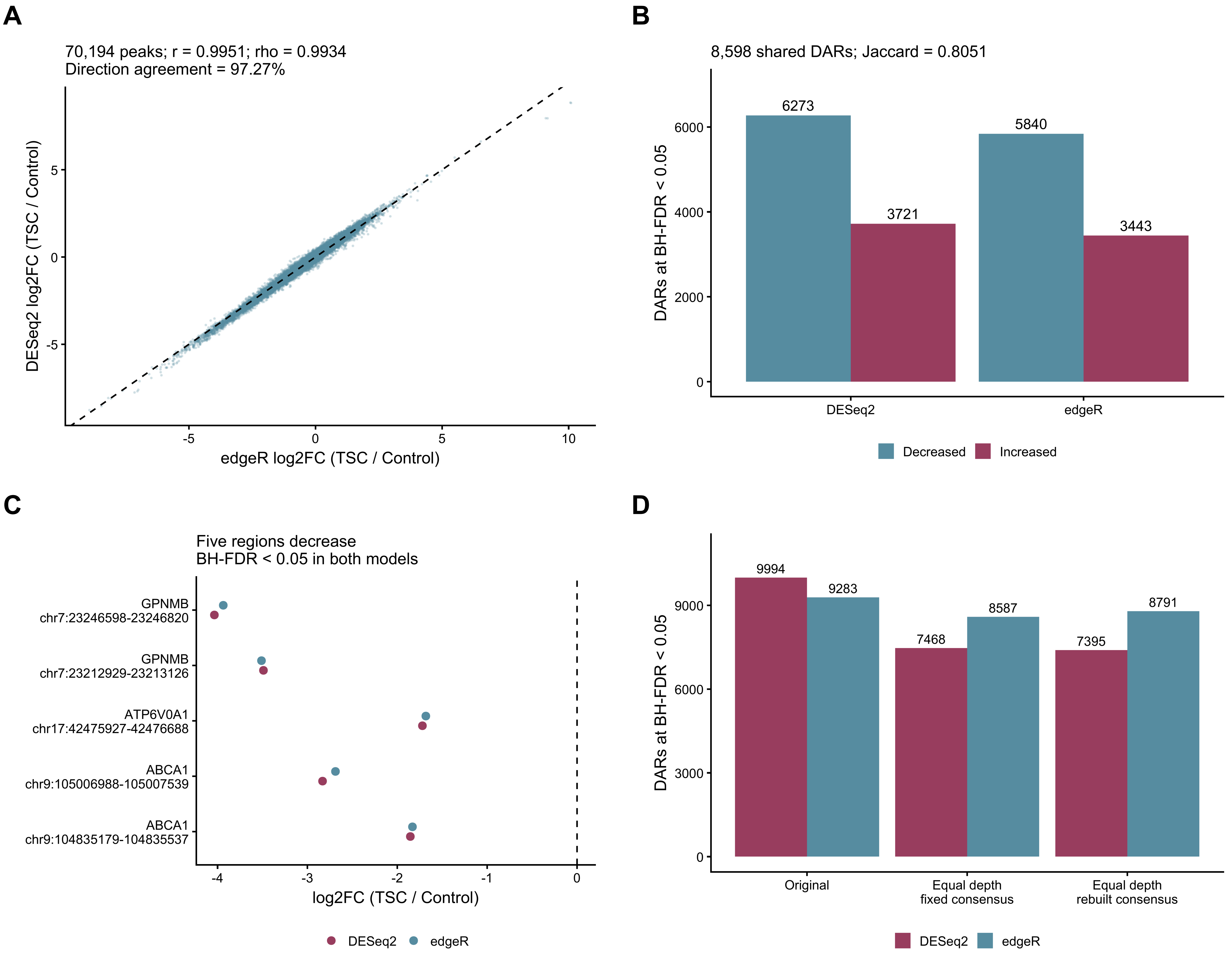

Figure legend

(A) edgeR and DESeq2 log2 fold changes across the same 70,194 tested peaks in the primary four-donor microglial population (two Control and two TSC donors; 6,040 nuclei). Correlations and sign agreement use all finite paired coefficients, without significance filtering. The dashed line indicates equality. (B) Increased and decreased DAR counts at BH-FDR < 0.05, without an additional fold-change cutoff; 8,598 DARs are shared. (C) Model coefficients for the five GREAT-predicted candidate regions; all have negative effects and BH-FDR < 0.05 in both models. (D) DAR counts under the original, equal-depth fixed-consensus, and equal-depth rebuilt-consensus scenarios. The first two test 70,194 peaks and the rebuilt scenario tests 57,883 peaks; direct set overlap is only interpreted within each scenario. Shared donors and data make this a sensitivity analysis, not independent validation. Full coefficients and FDR values are provided in Supplementary Table S8.
